# NETs‐CD44‐IL‐17A Feedback Loop Drives Th17‐Mediated Inflammation in Behçet's Uveitis

**DOI:** 10.1002/advs.202411524

**Published:** 2025-02-27

**Authors:** Yi Wu, Kang Ning, Zhaohao Huang, Binyao Chen, Junjie Chen, Yingying Wen, Jian Bu, Han Hong, Qiaorong Chen, Zhuoqi Zhang, Renbing Jia, Wenru Su

**Affiliations:** ^1^ State Key Laboratory of Ophthalmology Zhongshan Ophthalmic Center Sun Yat‐sen University Guangdong Provincial Key Laboratory of Ophthalmology and Visual Science Guangdong Provincial Clinical Research Center for Ocular Diseases Guangzhou 510060 China; ^2^ Department of Head and Neck Surgery Sun Yat‐sen University Cancer Center Guangzhou 510050 China; ^3^ State Key Laboratory of Oncology in Southern China Collaborative Innovation Center for Cancer Medicine Guangzhou 510050 China; ^4^ Bengbu Medical University Bengbu Anhui 233030 China; ^5^ Zhongshan School of Medicine Sun Yat‐sen University Guangzhou 510080 China; ^6^ Department of Ophthalmology Shanghai Key Laboratory of Orbital Diseases and Ocular Oncology Shanghai Ninth People's Hospital Shanghai Jiao Tong University School of Medicine Shanghai 200011 China

**Keywords:** Behçet's uveitis, CD44, IL‐17A, neutrophil extracellular traps, positive feedback loops

## Abstract

Behçet's uveitis (BU) is a severe ocular manifestation of Behçet's disease, typically accompanied by abnormal neutrophil infiltration and hyperactivation. However, the underlying causes of excessive neutrophil extracellular traps (NETs) production and mechanisms by which NETs contribute to the pathogenesis of BU remain incompletely understood. Neutrophils from BU patients exhibit a higher propensity for NETs release compared to healthy controls. In the experimental autoimmune uveitis (EAU), neutrophils are observed to exert pro‐inflammatory effects through NETs. Clearing NETs can inhibit T helper 17 (Th17) cell differentiation and significantly alleviate EAU symptoms. In vivo and in vitro experiments demonstrate neutralizing IL‐17A markedly reducing neutrophil infiltration and NETs formation in EAU. Single‐cell RNA sequencing confirms that CD44 plays a key role in mediating interactions between NETs and Th17 cells. Antagonizing CD44 inhibits the proportion of Th17 cells and NETs formation. Multiplex immunofluorescence and cell communication analyses further demonstrate interactions and colocalization between NETs and CD44^high^CD4^+^T cells in EAU. NETs induce Th17 differentiation via upregulating CD44, and in turn, Th17 cells secrete IL‐17A to recruit neutrophils and promote NETs formation. Interrupting NETs‐CD44‐IL‐17A feedback loop may be a potential therapeutic target for BU.

## Graphical abstract

In experimental autoimmune uveitis (EAU), neutrophils interact with T helper 17 (Th17) cells through neutrophil extracellular traps (NETs) to exert a pro‐inflammatory effect. In vitro and in vivo experiments identify that NETs promote IL‐17A secretion in Th17 cells via CD44, and increasing IL‐17A further induces more NETs formation. This NETs‐CD44‐IL‐17A feedback loop is a potential therapeutic target for uveitis.

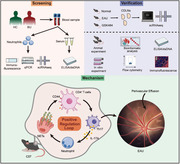

## 1 Introduction

Behçet's uveitis (BU) occurs in 60–80% of patients with Behçet's disease (BD) and is characterized by chronic relapsing bilateral nongranulomatous panuveitis and retinal vasculitis.^[^ ^1^, ^2^ ^]^ Persistent BU may cause complications such as cataracts, glaucoma, and destruction of the retina and choroid, and eventually lead to visual impairment or even blindness, severely impairing patients' daily activities and work capabilities.^[^ ^3^ ^]^ Studies have shown that CD4^+^ cells, particularly T helper 1 (Th1) and T helper 17 (Th17) cells, play a critical role in the onset and progression of BU.^[^ ^4^, ^5^, ^6^ ^]^ However, the exact pathogenesis of BU is considered to be complex, multifactorial, and not fully understood. Although corticosteroids and immunosuppressive agents were used to reduce inflammation and control BU‐related symptoms, their efficacy is limited and long‐term use may lead to various side effects.^[^ ^7^, ^8^ ^]^ Therefore, in‐depth research to identify the fundamental pathological mechanisms and novel core pathogenic factors of BU remains necessary to explore more specific, safe, and effective therapeutic strategies for clinical application.

Neutrophil extracellular traps (NETs) are net‐like structures released by activated neutrophils, consisting of decondensed chromatin, citrullinated histones, and various cellular proteins.^[^ ^9^ ^]^ The formation of NETs is a special form of programmed cell death termed netosis and involves a signaling cascade that includes the histone citrullination by the peptidylarginine deiminase 4 (PADI4), chromatin decondensation, and disintegration of the nuclear membrane.^[^ ^10^ ^]^ Increasing evidence highlights the crucial role of NETs as a link between innate and adaptive immune responses, which are strongly implicated in the pathogenesis of various diseases.^[^ ^11^, ^12^, ^13^ ^]^ In autoimmune diseases, NETs can contribute to the modification of autoantigens and subsequent presentation to the adaptive immune system, thereby regulating complex inflammatory responses that result in organ damage.^[^ ^12^ ^]^ For example, extensive NETs in the brain and spinal cord exacerbate the severity of autoimmune encephalomyelitis,^[^ ^14^ ^]^ and their accumulation in joints causes bone erosion associated with rheumatoid arthritis.^[^ ^15^ ^]^

Recent studies demonstrated the involvement of neutrophil activation and netosis in the onset and progression of BD, clearly establishing the clinical significance of NETs.^[^ ^16^, ^17^ ^]^ Joncour et al. reported that, compared to healthy control (HC), BD patients exhibit increased activation markers on neutrophils, enhanced production of reactive oxygen species, and upregulated netosis.^[^ ^16^ ^]^ Additionally, patients with BD have higher serum levels of NETs‐related markers, which correlate with local vasculitis and thrombosis.^[^ ^17^ ^]^ However, the pathological mechanisms underlying the extensive netosis and their involvement in the pathogenesis of BU remain elusive. To date, only a few studies have examined the role of exosomes and proteins such as IL‐8 in netosis within the experimental autoimmune uveitis (EAU) mouse model.^[^ ^18^, ^19^ ^]^ The reasons for the high accumulation of neutrophils and NETs in patients with BU, as well as the mechanisms by which NETs and CD4^+^ T cells cooperate to cause EAU, still need to be further elucidated.

In this study, we report that BU patients exhibit significantly elevated levels of NETs compared to HC. Using EAU model, we demonstrated that GSK484‐mediated blockade of netosis effectively alleviated retinopathy and pathological inflammatory cell infiltration. Both in vivo and in vitro experiments indicate a positive feedback loop between NETs and Th17, in which NETs promote Th17 differentiation and pathogenicity, and in turn, recruit more neutrophils and trigger NETs. Single‐cell sequencing (scRNA‐seq) revealed that the CD44 of CD4^+^ T cells acts as a potential target molecule of NETs. Antagonizing CD44 interrupts the NETs‐IL‐17A feedback loop in EAU mice. These findings suggest that targeting the NETs‐CD44‐IL‐17A positive feedback loop may be a novel therapeutic strategy for the treatment of BU.

## 2 Results

### 2.1 BU Patients Exhibit Elevated Levels of NETs Compared to HC

**Figure 1:**
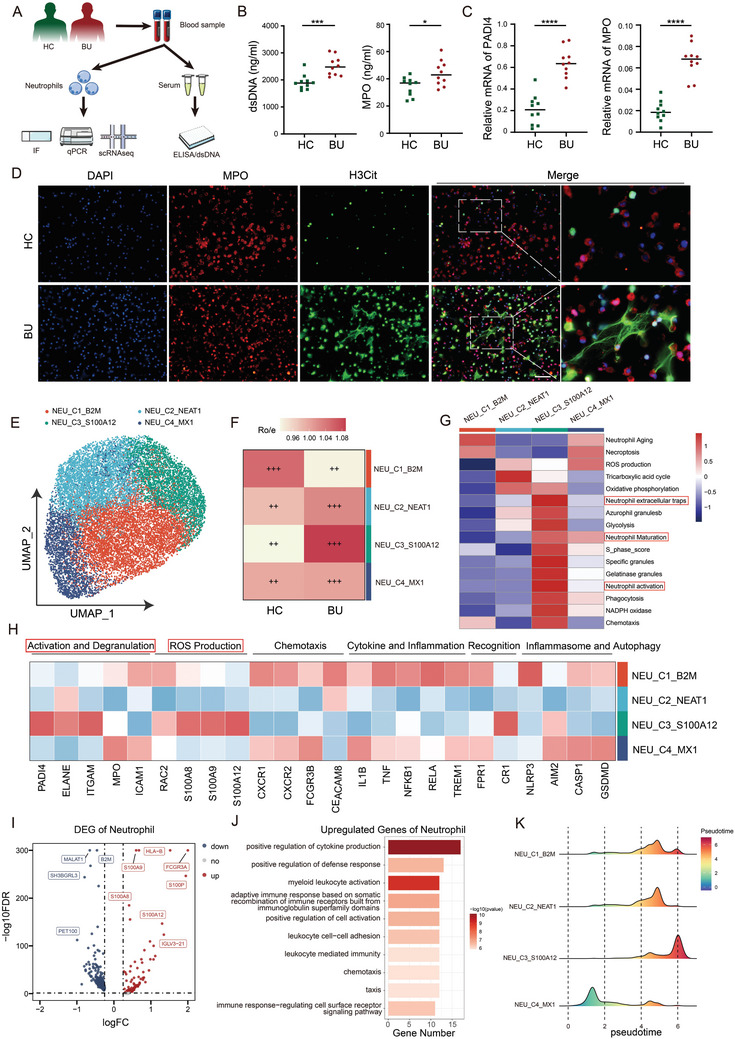
Elevated expression levels of NETs in BU patients. A) Schematic diagram of patient sample collection and experimental design. B) Expression levels of serum dsDNA and MPO in HC and BU patients (*n = *10 per group). C,D) Expression levels of NETs‐related genes and proteins in neutrophils of HC and BU patients detected by qPCR and IF. Scale bars, 50 µm. E) UMAP plot of scRNA‐seq subset analysis of neutrophils in HC and BU patients. F) Tissue preference analysis of neutrophil subsets from scRNA‐seq in HC and BU patients. G) GSVA pathway analysis of neutrophil subsets from scRNA‐seq. H) Functional gene display of neutrophil subsets from scRNA‐seq. I) Volcano plot of DEGs in neutrophils of HC and BU patients. J) GO pathway analysis of DEGs in neutrophils of HC and BU patients. K) Pseudotime analysis ridge plot of neutrophil subsets from scRNA‐seq. Significance in B‐C was determined using unpaired two‐tailed Student's *t*‐tests, *p < 0.05, **p < 0.01, ***p < 0.001, ****p < 0.0001.

Blood samples were collected from 10 pairs of BU patients and HC to isolate serum and neutrophils. Immunofluorescence (IF), real‐time quantitative polymerase chain reaction (qPCR), scRNA‐seq, and enzyme‐linked immunosorbent assay (ELISA) were conducted to validate the levels of NETs from HC and BU patients (**Figure** **1**A). Double‐stranded DNA (dsDNA) and myeloperoxidase (MPO) are serum markers of NETs, and higher levels of dsDNA and MPO were observed in BU compared to HC (Figure 1B). RNA extracted from neutrophils was subjected to qPCR, revealing elevated expression levels of NETs‐related genes *PADI4* and *MPO* in BU patients (Figure 1C). IF of isolated neutrophils also indicated that more NETs were formed in BU patients (Figure 1D).

ScRNA‐seq analysis was performed on enriched neutrophils from peripheral blood mononuclear cells (PBMC) of HC and BU patients, identifying four neutrophil subsets: NEU_C1_B2M, NEU_C2_NEAT1, NEU_C3_S100A12, and NEU_C4_MX1 (Figure 1E, Figure S1A,B, Supporting Information). Tissue preference analysis indicated heterogeneity among all neutrophil subsets in HC and BU patients, with the NEU_C3_S100A12 subset being highly enriched in BU patients (Figure 1F). Gene set variation analysis (GSVA) pathway and marker gene analysis of all neutrophil subsets revealed that upregulated pathways and genes of NEU_C3_S100A12 were enriched in neutrophil maturation, activation, reactive oxygen species production, and NETs formation (Figure 1G,H). Differentially expressed genes (DEGs) analysis of the neutrophil transcriptome revealed the upregulation of inflammation‐related genes such as *FCGR3A* and S100 family (S100A12, S100A9, S100A8, and S100P) in BU patients (Figure 1I, Figure S1C, Supporting Information). Gene ontology (GO) pathway analysis indicated the upregulation of pathways involved in cytokine production, myeloid leukocyte activation, and chemotaxis in BU patients (Figure 1J, Figure S1D, Supporting Information). Pseudotime analysis revealed that the expression levels and functions of NETs‐related genes (such as *PADI4*, *ITGAM*, and *RAC2*) gradually increased along the pseudotime trajectory, while NEU_C3_S100A12 was more enriched at the end of pseudotime (Figure 1K, Figures S1E and S2A,B, Supporting Information).

The results indicate that BU patients exhibited higher levels of NETs formation and that their neutrophils have a greater propensity to produce NETs. Neutrophils especially NEU_C3_S100A12 play a significant role in the pathogenesis of BU.

### 2.2 Clearing of NETs Alleviates the Onset of EAU

**Figure 2:**
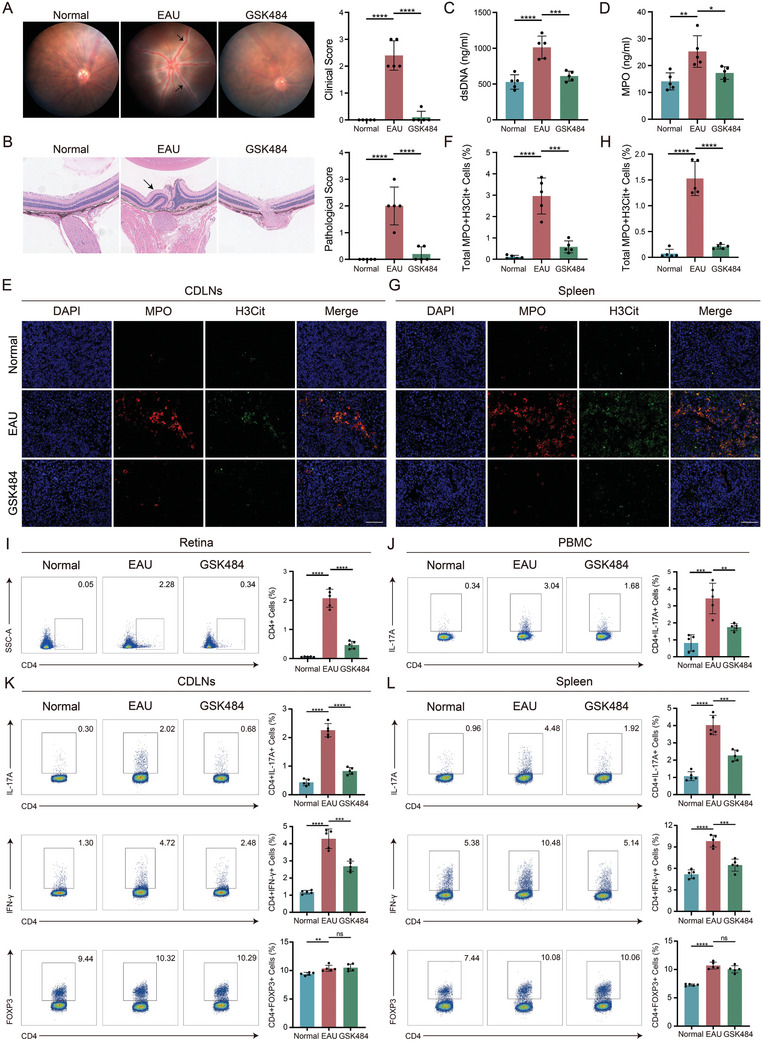
Inhibition of NETs levels alleviates severity of EAU. A,B) Fundus images, clinical scores, H&E staining, and pathological scores of the normal, EAU, and GSK484 groups (*n = *5 per group). C,D) Serum levels of dsDNA and MPO in the normal, EAU, and GSK484 groups (*n = *5 per group). E,F) IF expression levels of NETs‐related proteins in the CDLNs of normal, EAU, and GSK484 groups (*n = *5 per group). Scale bars, 50 µm. G,H) IF expression levels of NETs‐related proteins in the spleen of normal, EAU, and GSK484 groups (*n = *5 per group). Scale bars, 50 µm. I) FCM analysis of the proportion of infiltrating CD4^+^ T cells in the retina (*n = *5 per group). J) FCM analysis of IL‐17A in CD4^+^ T cells derived from PBMC (*n = *5 per group). K,L) FCM analysis of IL‐17A, IFN‐γ, and FOXP3 expression in CD4^+^ T cells derived from CDLNs and spleen of the normal, EAU, and GSK484 groups (*n = *5 per group). Significance in A,B was determined using the Kruskal–Wallis test. Significance in C‐L was determined using one‐way ANOVA and Bonferroni multiple comparison test. Results are presented as mean ± SEM, with ns indicating no significant difference, *p < 0.05, **p < 0.01, ***p < 0.001, ****p < 0.0001.

The PADI4 inhibitor GSK484 could prevent netosis by reducing histone citrullination, a key process required for chromatin decondensation and DNA release.^[^ ^20^ ^]^ To explore the role of NETs in BU, we immunized C57BL/6J mice with IRBP_1‐20_ to establish a GSK484‐treated EAU group and an EAU control group. Fundus and pathology scores indicated that the clearance of NETs with GSK484 significantly alleviated the symptoms of EAU (**Figure** **2**A,B). Serum analysis from the mice revealed that serum levels of dsDNA and MPO were significantly higher in the EAU mouse model compared to the normal group, but treatment with GSK484 reduced these levels (Figure 2C,D).

Cervical drain lymph nodes (CDLNs) are considered the draining lymph nodes of the eye and are critical sites for immune cell interactions, antigen presentation, and immune cell activation, which are closely related to the development of BU.^[^ ^21^ ^]^ IF analysis of CDLNs and spleen samples revealed that the EAU group produced more NETs compared to the normal and GSK484 groups (Figure 2E–H). This finding is consistent with previous observations in BU patients^[^ ^19^ ^]^ and confirms that GSK484 successfully reduced elevated NETs in the EAU model. CD4^+^ T cells, particularly the increase of Th1/17 cells and the decrease of Tregs, are closely associated with the development of EAU. Flow cytometry (FCM) revealed that clearing NETs significantly reduced the number of Th17 cells in the PBMC and CD4^+^ T cell infiltration in the retina (Figure 2I,J). GSK484 treatment changed the distribution of CD4^+^ T cells, reducing the proportions of Th17 (CD4^+^IL‐17A^+^) and Th1 (CD4^+^IFNγ^+^) cells without affecting the levels of Treg (CD4^+^FOXP3^+^) (Figure 2K,L, Figure S6A, Supporting Information). The transcription factors (RORγt, STAT3, IRF4) of CD4^+^ were also upregulated after GSK484 treatment (Figure S4C, Supporting Information). Padi4‐KO mice are unable to generate NETs normally, and repeated experiments in PADI4‐KO mice obtained results similar to those observed with GSK484 treatment (Figure S5A–F, Supporting Information). Overall, clearing NETs in the EAU mouse model significantly alleviates fundus inflammation, primarily by reducing Th1 and Th17 differentiation.

### 2.3 Positive Feedback Regulation Between NETs and IL‐17A

**Figure 3:**
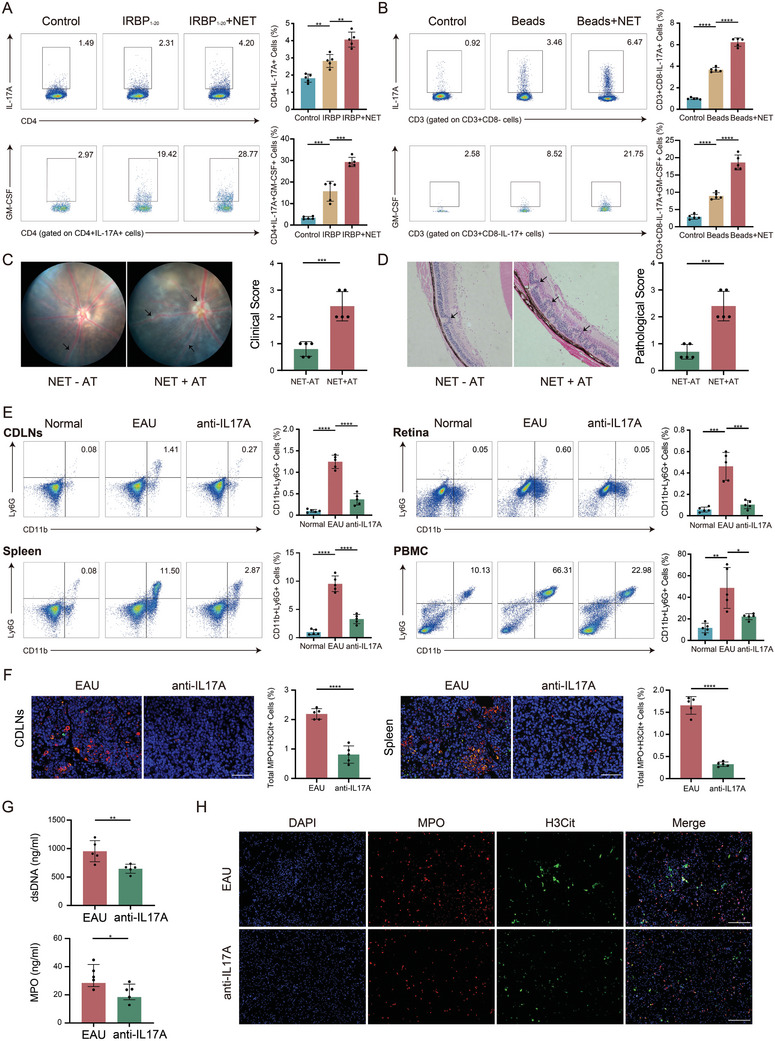
Interaction between NETs and IL‐17A. A,B) In vitro experiments show that NETs promote the expression of IL‐17A and GM‐CSF in mouse (A) and human (B) CD4^+^ T cells (*n = *5 per group). C,D) Fundus images, clinical scores, H&E staining, and pathological scores in different groups from mouse adoptive transfer experiments (*n = *5 per group). (E) FCM analysis of neutrophil infiltration in CDLNs, spleen, retina, and PBMC between normal, EAU, and anti‐IL‐17A treatment groups (*n = *5 per group). F) IF assessment of NETs formation in CDLNs, spleen, retina, and PBMC of two groups (*n = *5 per group). Scale bars, 50 µm. G) Serum levels of dsDNA and MPO of two groups (*n = *5 per group). H) IF evaluation of NETs formation of bone marrow neutrophils of EAU control and anti‐IL‐17A treatment group. Scale bars, 200 µm. Significance in A,B, E–G was determined using one‐way ANOVA and Bonferroni multiple comparison test. Significance in C‐D was determined using Mann–Whitney Test. Results are presented as mean ± SEM, *p < 0.05, **p < 0.01, ***p < 0.001, ****p < 0.0001.

Neutrophils were isolated from EAU mice and BD patients, enriched and induced to form NETs in vitro, and then co‐cultured with CDLNs and PBMC. FCM analysis revealed that the proportion of Th17 cells increased in CD4^+^ T cells co‐cultured with NETs, and these Th17 cells exhibited increased secretion of GM‐CSF (**Figure** **3**A,B). In an adoptive transfer experiment, immune cells from EAU mice that had been co‐cultured with NETs in vitro were transferred into normal mice (Figure S3, Supporting Information). The results showed that the immune cells co‐cultured with NETs enhanced the EAU symptoms in the recipient mice (Figure 3C,D).

As an important immunomodulatory factor, the effect of IL‐17A on neutrophil function was further investigated. In the EAU model, treatment with anti‐IL‐17A resulted in a significant reduction in the proportion of infiltrating neutrophils (CD11b^+^Ly6G^+^) in the CDLNs, spleen, PBMC, and retina as detected by FCM and immunohistochemistry (IHC) (Figure 3E, Figure S7A, Supporting Information). IF analysis also indicated that the levels of NETs formation in the CDLNs and spleen were significantly decreased following anti‐IL‐17A treatment (Figure 3F; Figure S7B, Supporting Information). Serum analysis showed that levels of dsDNA and MPO in serum were lower in the anti‐IL‐17A group (Figure 3G). In vitro culture of neutrophils isolated from mouse bone marrow demonstrated that inhibiting IL‐17A reduced the formation of NETs (Figure 3H). These findings suggest that NETs can promote the generation of Th17 cells, and Th17 cells can further enhance neutrophil recruitment to form more NETs. This also indicates a pathological positive feedback loop between NETs and Th17 cells in the EAU mouse model.

### 2.4 Changes in the Immune Microenvironment Following NETs Clearance Revealed by scRNA‐seq

**Figure 4:**
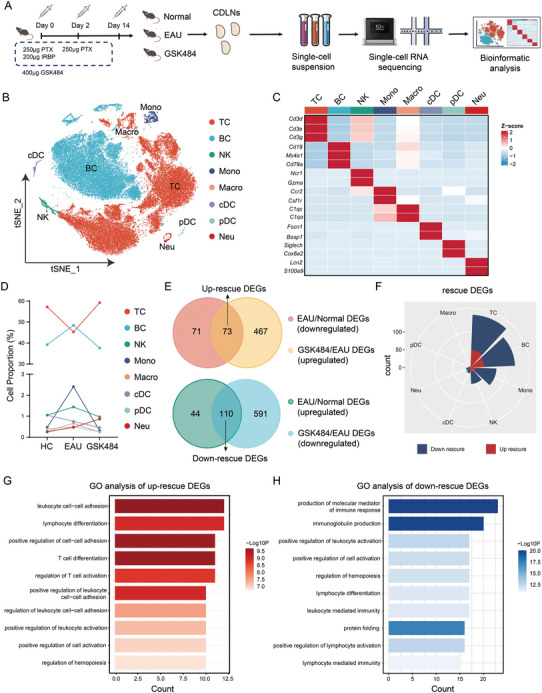
ScRNA‐seq analysis of CDLN cells in different experimental groups. A) Schematic diagram of mouse sample collection and experimental design. B) tSNE plots showing the classification of individual cells derived from scRNA‐seq of CDLN cells from all mice groups. C) Heatmap representation of marker expression profiling used for cell identification in CDLN cells from all mice groups. D) Proportional comparison of cell compositions across various groups of CDLN cells. E,F) Volcano plots depicting the differential expression analysis of CDLN cell subsets in the EAU versus HC, and GSK484 versus EAU comparisons. G,H) GO pathway analyses of upregulated and downregulated DEGs in CDLN cells.

ScRNA‐seq was performed on CDLNs collected from mice in the normal, EAU, and GSK484 groups (**Figures** **4**A, S8A, Supporting Information). The analysis identified eight major cell populations within the CDLNs: T cells (TC), B cells (BC), natural killer cells (NK), monocytes (Mono), macrophages (Macro), classical dendritic cells (cDC), plasmacytoid dendritic cells (pDC) and neutrophils (Neu) (Figure 4B–D, Figure S8B, Supporting Information). We focus on the subset of EAU‐DEGs that were rescued by GSK484, termed “rescue DEGs”, including 73 upregulated rescue DEGs and 110 downregulated rescue DEGs (Figure 4E). Most of these rescue DEGs were concentrated in T cells and B cells (Figure 4F). GSK484 treatment effectively inhibited the expression of several inflammatory genes across different cell types in EAU (Figure S8C,D, Supporting Information). GO pathway analysis of the rescue DEGs indicated that up‐rescue DEGs were predominantly associated with T cell differentiation (Figure 4G), whereas down‐rescue DEGs were involved in protein folding, cytokine secretion, and T cell activation (Figure 4H). GSVA analysis showed that NETs formation by neutrophils was upregulated in EAU, while GSK484 inhibited various functions of neutrophils (Figure S8E,F, Supporting Information). The cell proportion and signaling pathways of B cell subsets also significantly changed in EAU mice after GSK484 treatment (Figure S9A–E, Supporting Information).

**Figure 5:**
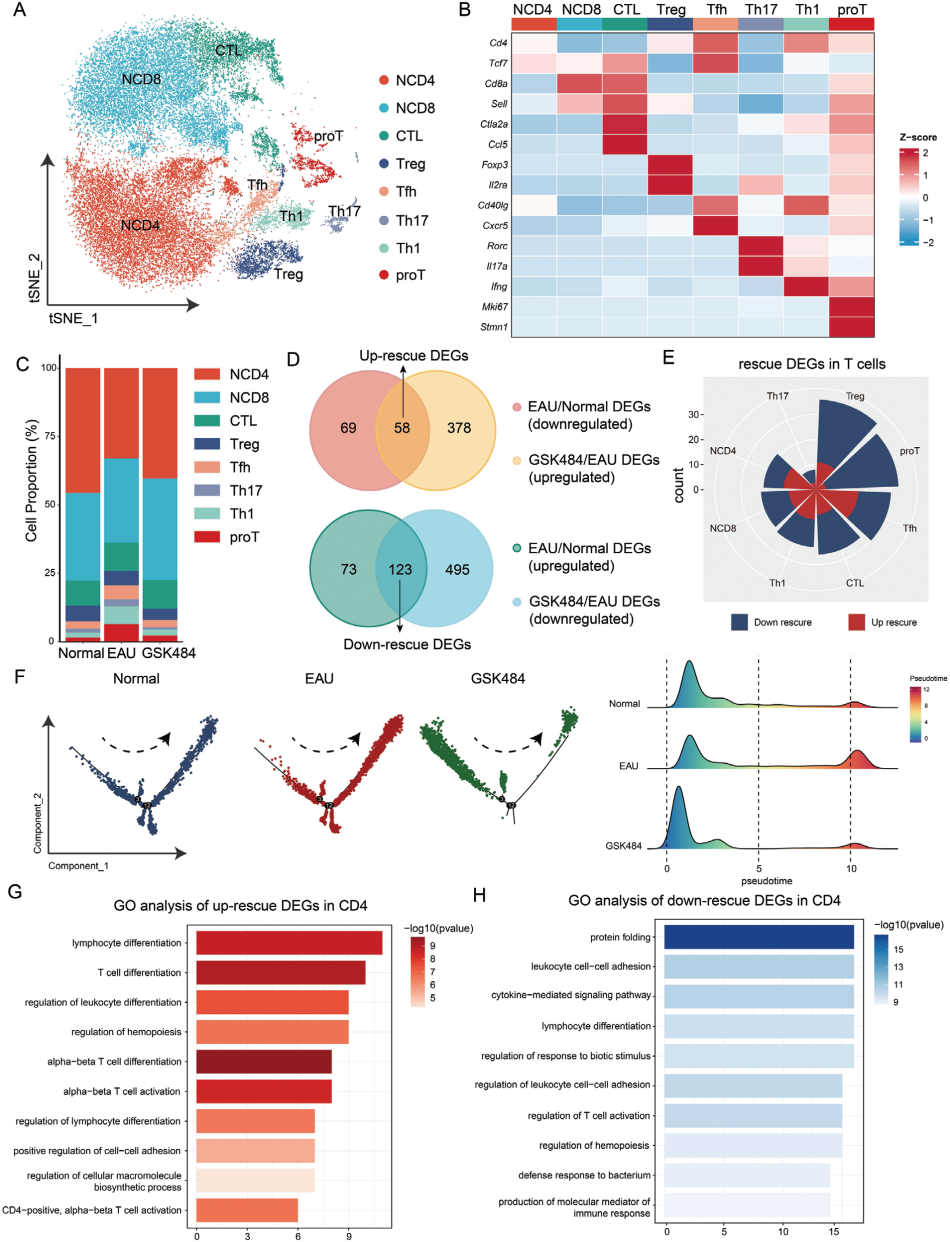
ScRNA‐seq analysis of T cell subsets across various groups. A) tSNE plots illustrating the cell identification results for T cell subsets from all mice groups. B) Heatmap detailing the expression of identification markers in T cell subsets from all mice groups. C) Comparison of the composition ratios of T cell subsets across different groups. D,E) Analysis of the number and distribution of DEGs in T cells among various cell clusters. F) Pseudotime analysis of T cell subsets across different groups. G,H) GO pathway analysis of upregulated and downregulated DEGs within CD4^+^ T cells.

After subgroup analysis of T cells, eight major subsets were identified: naïve CD4^+^ T cells (NCD4), naïve CD8^+^ T cells (NCD8), cytotoxic T lymphocytes (CTL), regulatory T cells (Treg), follicular helper T cells (Tfh), Th17 cells, Th1 cells, and proliferative T cells (proT) (**Figure** **5**A,B, Figure S10A,B, Supporting Information). Following GSK484 treatment, the proportions of Tfh, Th17, Th1, and proT cells decreased in EAU (Figure 5C). DEGs analysis of T cells revealed 58 up‐rescue DEGs and 123 down‐rescue DEGs, with the majority of DEGs concentrated in CD4^+^ T cell subsets (Figure 5D,E, Figure S10C,D, Supporting Information). Pseudotime analysis of CD4^+^ T cells indicated that in the EAU group, CD4^+^ T cells were predominantly at the terminal stage of pseudotime, whereas GSK484‐mediated NETs clearance reversed this trend (Figure 5F, Figures S10E–G and S11A–D, Supporting Information). GO pathway analysis results were consistent with the analysis of the total CDLNs cell population, showing that GSK484 inhibited protein folding and cytokine secretion functions of active T cells in EAU (Figure 5G,H). These findings identified again that NETs clearance can suppress autoimmune functions by inhibiting CD4^+^ T cells, thereby reducing the formation of Th17 cells.

### 2.5 Inhibition of CD44 disrupts the positive feedback loop between NETs and IL‐17A

**Figure 6:**
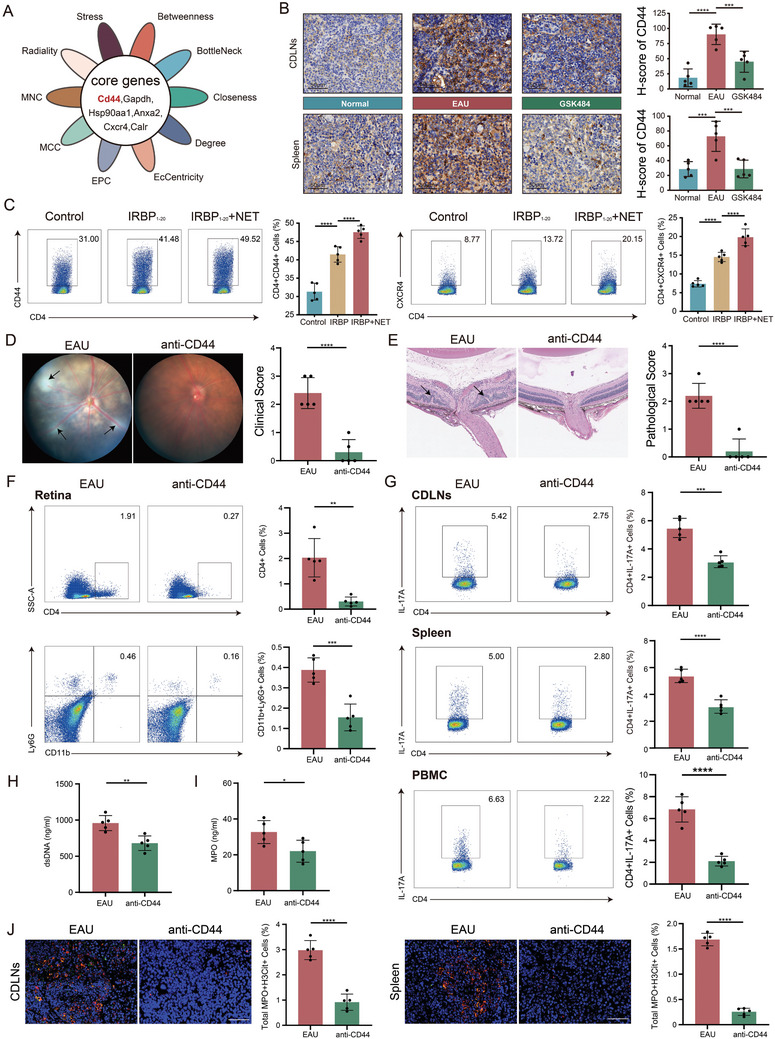
CD44 mediates the positive feedback between IL‐17A and NETs. A) Venn diagram of core genes calculated using different algorithms. B) IHC assessment of CD44 expression levels in different groups (*n = *5 per group). C) FCM analysis of CD44 and Cxcr4 expression levels in in vitro cultures (*n = *5 per group). D,E) Fundus images, clinical scores, H&E staining, and pathological scores of EAU mouse models treated with anti‐CD44 (*n = *5 per group). F) Proportion of infiltrating CD4^+^ T cells and neutrophil (CD11b^+^ LY6G^+^) in the retina of EAU and anti‐CD44 groups (*n = *5 per group). G) Expression levels of IL‐17A in CD4^+^ T cells derived from CDLNs, spleen, and PBMC of EAU and anti‐CD44 groups (*n = *5 per group). H,I) Serum levels of dsDNA and MPO in two groups (*n = *5 per group). J) IF assessment of NETs levels in CDLNs and spleen of EAU and anti‐CD44 groups (*n = *5 per group). Scale bars, 50 µm. Significance in B,C was determined using one‐way ANOVA and Bonferroni multiple comparison test. Significance in D,E was determined using Mann–Whitney Test. Significance in F‐J was determined using unpaired two‐tailed Student's *t*‐tests. Results are presented as mean ± SEM, *p < 0.05, **p < 0.01, ***p < 0.001, ****p < 0.0001.

Using different hub gene algorithms, we identified six core genes from the top 20 hub genes of down‐rescue DEGs in CD4^+^ T cells: *Cd44, Gapdh, Hsp90aa, Anxa2, Cxcr4, and Calr* using the cytoHubba plugin with ten algorithms (**Figures** **6**A, S12A–J, Supporting Information). These genes gradually increased along the pseudotime trajectory and were upregulated in the EAU group, but GSK484 inhibited their upregulation in all CD4^+^ T cell subsets (Figures S13A–G and S11E, Supporting Information). Among these, the membrane receptor CD44 may regulate the differentiation of CD4^+^ T cells through NETs,^[^ ^22^ ^]^ while CXCR4 is an important chemokine receptor on T cells, particularly in the context of autoimmune diseases.^[^ ^23^, ^24^ ^]^ IHC scoring validated that CD44 expression is upregulated in the EAU group, and GSK484 can inhibit the overexpression of CD44 in the EAU group (Figure 6B). In vitro and in vivo experiments also demonstrated that NETs can increase the expression of CD44 and CXCR4 on CD4^+^ T cells, while GSK484 treatment and PADI4 knockout in mice have opposing effects (Figure 6C; Figures S4A,B and S5G,H, Supporting Information). In vitro and in vivo experiments showed that inhibiting CD44 can ameliorate EAU symptoms in the EAU model (Figure 6D,E). Inhibiting CD44 significantly reduced the proportion of infiltrating CD4^+^ T cells and neutrophils (CD11b^+^ LY6G^+^) in the retina (Figure 6F). The proportion of Th17 (CD4^+^IL17A^+^) and Th1 (CD4^+^IFNγ^+^) cells decreased without affecting the levels of Treg in anti‐CD44 group (Figure 6G, Figure S6B,C, Supporting Information). Treatment with anti‐CD44 also decreased the levels of NETs in EAU, as evidenced by a significant reduction in serum dsDNA and MPO levels (Figure 6H,I), and a decreased proportion of MPO^+^H3Cit^+^ cells in the CDLNs and spleen (Figure 6J; Figure S7C, Supporting Information). In vitro experiments were conducted where CD4^+^ T cells co‐cultured with untreated NETs, Proteinase K‐treated NETs, or DNaseI‐treated NETs, and it was found the degradation of protein components significantly enhanced the expression of IL‐17A and CD44 in CD4^+^ T cells, while degradation of DNA component had no significant effect on these markers (Figure S4D, Supporting Information).

In summary, CD44 is a key molecule in the interaction between NETs and Th17 cells. NETs regulate Th17 differentiation by upregulating CD44 expression in CD4^+^ T cells, thereby promoting the secretion of IL‐17A and driving the formation of a pathological NETs‐CD44‐IL‐17A positive feedback loop.

### 2.6 Interaction and Colocalization of Neutrophils with CD44^high^CD4^+^ T Cells

**Figure 7:**
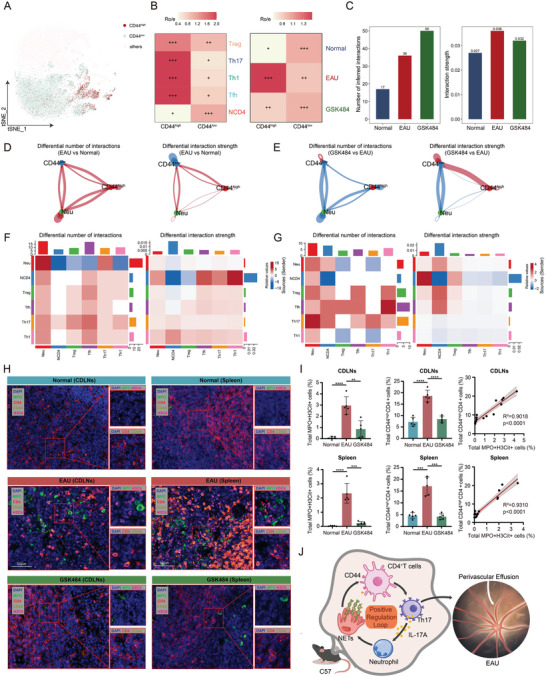
Neutrophils interact with CD44^high^CD4^+^ cells through NETs. A) tSNE distribution plot of CD44^high^CD4^+^ T cells. B) Tissue preference analysis of CD44^high^CD4^+^ T cells versus CD44^low^CD4^+^ T cells. C) Bar chart of cell communication quantity and intensity across normal, EAU, and GSK484 groups. D,E) Interaction network and heatmap of cell communication quantity and intensity between CD44^low^CD4^+^ T cells, CD44^high^CD4^+^ T cells, and neutrophils in EAU versus normal group and in GSK484 versus EAU group. F,G) Interaction network and heatmap of cell communication quantity and intensity between CD4^+^ T cell subsets and neutrophils in EAU versus normal group and in GSK484 versus EAU group. H) MIF staining of spleen and CDLNs in different groups. I) Quantification of MIF staining in spleen and lymph nodes across different groups (*n = *5 per group). J) Interaction model between neutrophils and CD44^high^CD4^+^ cells. Significance in I was determined using one‐way ANOVA followed by Bonferroni multiple comparison test and Spearman correlation test. Results are presented as mean ± SEM, *p < 0.05, **p < 0.01, ***p < 0.001, ****p < 0.0001.

CD44^high^CD4^+^ T cells were defined as CD4^+^ T cells with CD44 gene expression greater than 0 in single‐cell sequencing (**Figure** **7**A). Tissue preference analysis indicated that CD44^high^CD4^+^ T cells were enriched in EAU mice, primarily consisting of Th1, Th17, and Tfh cells (Figure 7B, Figure S11E, Supporting Information). Cell communication analysis revealed increased cell communication intensity of CD4^+^ T cells in EAU (Figure 7C). A comparative analysis of cell communication quantity and intensity across different groups showed the communication intensity between neutrophils and CD44^high^CD4^+^ T cells increased in the EAU group compared to the normal group, and the clearance of NETs by GSK484 significantly reversed this process (Figure 7D,E, Figure S14A, Supporting Information). This suggested that neutrophils primarily interact with CD44^high^CD4^+^ T cells through NETs. Similarly, the enhanced interaction between Th17 and neutrophil in EAU was also reversed after GSK484 treatment, especially in the differential number of interactions (Figure 7F,G; Figure S14B, Supporting Information). The cell‐cell communication signal analysis found that the Lgals9‐Cd44 interaction may play a functional role in the interaction between neutrophils and CD4^+^ T cells (Figure S14C–H, Supporting Information). Multiplex immunofluorescence (MIF) staining demonstrated colocalization of NETs (MPO^+^H3Cit^+^) with CD44^high^CD4^+^ T cells in the EAU group (Figure 7H, Figure S15A,B, Supporting Information). Treatment with GSK484 reversed the increase in NETs and CD44^high^CD4^+^ T cells in the EAU group, and a positive correlation was observed between NETs and CD44^high^CD4^+^ T cells (Figure 7I). In summary, neutrophils can form NETs that promote the formation of CD44^high^CD4^+^ T cells, while CD44^high^CD4^+^ T cells induce more neutrophil infiltration and NETs formation through IL‐17A. This pathological NETs‐CD44‐IL‐17A positive feedback loop leads to the onset of EAU (Figure 7J).

## 3 Discussion

BD is a systemic vasculitis characterized by recurrent orogenital ulceration and BU. BU is an inflammatory disorder that manifests with massive retinal vascular leakage and inflamed lesions, predominantly driven by Th17/Treg immune imbalances, and eventually leads to visual impairment and blindness.^[^ ^1^, ^2^ ^]^ Identifying novel therapeutic targets and exploration of core pathogenic factors and associated pathways in the pathogenesis of BU are imperative. Therefore, we conducted relevant research and summarized the primary findings as follows: 1) Netosis is elevated in the peripheral circulation of BU patients and correlates with CD4^+^ T cell‐mediated pathogenesis. 2) GSK484‐mediated blockade of Netosis effectively alleviated retinopathy and pathological inflammatory cell infiltration in EAU mice. 3) GSK484 treatment in EAU mice reprogrammed the cell proportion and rescued EAU‐induced Th17/Treg immune imbalance, inhibiting Th17 differentiation and pathogenicity without affecting Treg quantity. 4) In vitro generated NETs co‐cultured with both mouse CDLN cells and human PBMC promoted Th17 differentiation and pathogenicity. 5) IL‐17A recruits neutrophils and triggers Netosis in EAU, which can be mitigated by neutralizing IL‐17A. 6) CD44 acts as a potential target molecule of NETs to regulate Th17 differentiation and pathogenicity and contribute to EAU pathogenesis. 7) NETs‐CD44‐IL‐17A positive feedback loop is associated with BU progression and contributes to human Th17 cell differentiation.

NETs were initially recognized as a defensive mechanism by which neutrophils capture and kill microorganisms in response to pathogenic infections.^[^ ^9^, ^10^ ^]^ However, recent research has revealed that NETs play crucial roles in the pathological processes of both tumors and autoimmune diseases.^[^ ^25^ ^]^ There is also a potential pathological positive feedback mechanism between NETs and tumor cells.^[^ ^13^ ^]^ Studies have shown that tumors can induce NETs formation by secreting various tumor‐derived and infection‐related factors.^[^ ^26^, ^27^ ^]^ In turn, the deposition of NETs promotes tumor cell proliferation, immune suppression, and cancer‐associated thrombosis, further enhancing the formation of NETs‐inducing factors.^[^ ^28^, ^29^ ^]^ Additionally, NETs can exacerbate autoimmune diseases such as systemic lupus erythematosus and rheumatoid arthritis by exposing self‐antigens, promoting inflammatory responses, or directly damaging tissues.^[^ ^12^ ^]^ In‐depth research into NETs can provide a better understanding of the dual role of neutrophils in immune suppression (in tumors) and immune activation (in autoimmune diseases).

Our study found heterogeneity in neutrophils among BU patients, which is one of the reasons for the upregulation of NETs in these patients. Previous studies have also described similar abnormal neutrophil infiltration phenomena in BD patients.^[^ ^17^, ^30^, ^31^, ^32^ ^]^ Joncour et al. demonstrated that NETs and markers of NETs are elevated in patients with BD and contribute to the procoagulant state.^[^ ^17^ ^]^ In addition, Wang et al. discovered that the heterogeneity of neutrophil subsets between males and females is associated with the higher incidence and severity of BU in males.^[^ ^32^ ^]^ However, neutrophil activation and differentiation often depend on the induction of various cytokines, and the heterogeneity of neutrophils alone is insufficient to explain the complex and persistent inflammatory immune response in BU patients.^[^ ^30^ ^]^ A signaling network involving multiple cell types, including lymphocytes, endothelial cells, and platelets, may induce neutrophil infiltration and exert pathogenic effects through NETs.

The pathological positive feedback loop between NETs and Th17 cells provides a reasonable explanation for the extensive neutrophil infiltration and NETs production observed in BU patients. Previous research has largely focused on the unidirectional effects of various components within the microenvironment, but this approach is insufficient to explain the exceptionally high levels of NETs observed in BU.^[^ ^19^, ^33^, ^34^, ^35^, ^36^, ^37^ ^]^ The uncontrolled positive feedback loop generated by the interaction between neutrophils and Th17 cells is a critical factor driving this persistent and complex inflammatory response in BU. In this loop, abnormal CD4^+^ T cells or neutrophils could be the initiators of the pathological positive feedback. For example, inflammation triggered by trauma or infection may produce a large amount of NETs, promoting the exposure of self‐antigens and inducing the NETs‐CD44‐IL‐17A pathological feedback.^[^ ^38^ ^]^ Additionally, a specific neutrophil subset (NEU_C3_S100A12) observed in BU patients contributes to NETs formation and may also serve as a trigger for this cycle. The above results also indicate that controlling the factors that inducing NETs formation and inhibiting the NETs‐CD44‐IL‐17A pathological feedback loop are crucial for the early prevention of BU onset and progression.

The pathological positive feedback loop formed by the interaction between neutrophils and Th17 cells induces the involvement of various cellular subsets and factors in the development of BU. The interaction between neutrophils and CD4^+^ T cells is a complex process involving multiple cells and factors. IL‐17A plays a crucial role in the inflammatory response, as it can induce epithelial cells, fibroblasts, and other immune cells to secrete pro‐inflammatory cytokines such as IL‐6, IL‐8, and GM‐CSF, thereby promoting the recruitment and activation of neutrophils.^[^ ^19^, ^36^, ^39^, ^40^ ^]^ Additionally, components of NETs, such as DNA, H3Cit, and MPO, not only cause direct tissue damage but can also act as autoantigens, leading to abnormal activation of T cells and B cells.^[^ ^35^, ^41^, ^42^, ^43^ ^]^ Moreover, NETs can exacerbate inflammation by affecting macrophage polarization (e.g., M1 polarization) and increasing endothelial cell permeability.^[^ ^44^, ^45^, ^46^ ^]^ The interaction between neutrophils and Th17 cells is a key pathological mechanism in BU, triggering cellular crosstalk and a cytokine storm that leads to disease onset. Overall, preventing the excessive activation of neutrophils and the formation of NETs could help alleviate the inflammatory response and mitigate the symptoms of BU.

Although various drugs have shown that clearing NETs can alleviate EAU symptoms,^[^ ^16^, ^47^ ^]^ there are currently no NETs‐targeted drugs available in clinical practice. This may be due to the risks associated with directly targeting NETs, as it could impair their ability to clear bacteria and viruses, leading to excessive immunosuppression and associated side effects.^[^ ^48^, ^49^, ^50^ ^]^ Although the clearance of IL‐17A can reduce the formation of NETs, several clinical studies have shown that secukinumab (an IL‐17A inhibitor) has not achieved satisfactory efficacy in the treatment of uveitis.^[^ ^51^, ^52^, ^53^ ^]^ This is due to the presence of a negative feedback loop driven by IL‐24, which, after IL‐17A inhibition, promotes the expression of other inflammatory factors, such as GM‐CSF and IL‐24, in Th17 cells.^[^ ^54^ ^]^ Besides, the existence of autonomously activating the IL‐17R signaling pathway also contributes to the suboptimal efficacy of anti‐IL‐17A therapy.^[^ ^55^ ^]^ Therefore, directly targeting NETs and IL‐17A to control the formation of the NETs‐IL‐17A feedback loop may not be the most ideal strategy.

CD44 plays a crucial role in the pathological positive feedback loop between NETs and Th17 cells, making it a potential downstream therapeutic target. CD44 is a specific protein highly expressed in BU, associated with increased leukocyte adhesion and blood‐retinal barrier permeability.^[^ ^56^, ^57^ ^]^ Studies have shown a significant increase in the number of CD44^high^ Th17 cells in the retina, cervical lymph nodes, inguinal lymph nodes, and spleen.^[^ ^58^ ^]^ In experimental autoimmune encephalitomyelitis, CD44 expression is crucial for Th cell differentiation, with its absence inhibiting Th1/Th17 differentiation and enhancing Th2/regulatory T cell differentiation.^[^ ^59^ ^]^ Inhibiting CD44 can effectively suppress the abnormal activation of CD4^+^ T cells, reducing the recruitment and localization of immune cells at inflammation sites.^[^ ^60^, ^61^, ^62^ ^]^ Targeting CD44 represents a potential strategy to control the NETs‐IL‐17A feedback loop, as it can effectively regulate the production of Th17 cells and their associated inflammatory factors while preserving the essential functions of neutrophils in the body, thereby minimizing excessive side effects.

In summary, clinical specimens have demonstrated that neutrophil activation and NETs relase are elevated in BU patients. Combining scRNA‐seq with in vivo and in vitro experiments, we discovered that the NETs‐CD44‐IL‐17A pathological positive feedback loop contributes to the inflammatory environment in EAU and GSK484‐mediated blockade of Netosis can effectively reduce Th17 differentiation and pathogenicity. CD44 is a crucial intermediate target in the NETs‐CD44‐IL‐17A pathological feedback loop. IL‐17A promotes neutrophil recruitment and triggers NETs formation, while NETs induce Th17 cell differentiation and pathogenicity by upregulating CD44 expression. Targeting this pathological feedback loop is a potential therapeutic strategy for treating BU, and it may enhance early intervention, improve management, and better prognosis for BU patients.

## 4 Experimental Section

### Human Donors

Ten pairs of newly diagnosed and untreated active BU patients and HC matched for age and sex were recruited at the Zhongshan Ophthalmic Center, Sun Yat‐sen University, Guangzhou, China. The diagnosis of BU was made according to the criteria of the International Study Group for Behçet's disease (ICBD).^[^ ^63^ ^]^ This study protocol was reviewed and approved by the Medical Ethics Committee of the Zhongshan Ophthalmic Center (2020KYPJ124). Neutrophils from BU patients and HC were isolated for scRNA‐seq to explore neutrophil heterogeneity. This study protocol was reviewed and approved by the Medical Ethics Committee of the Zhongshan Ophthalmic Center in Guangzhou, and written informed consent was obtained from all participants. All individual data were recorded in Table S1, Supporting Information.

### Mice

Wild‐type female C57BL/6 J mice aged 6–8 weeks and weighing 18–25 g were procured from the Medical Laboratory Animal Center in Guangzhou, China. The PADI4‐KO mice and their matched negative control mice were obtained from Shanghai Model Organisms Center, Inc. in China. These mice were maintained under specific pathogen‐free conditions at a constant temperature of 21 ± 1 °C and a relative humidity of 60 ± 5%, with a 12 h light/dark cycle implemented. The animal experiments described herein were conducted in strict accordance with established guidelines for animal welfare and usage and were approved by the Institutional Animal Care Committee of the Zhongshan Ophthalmic Center at Sun Yat‐Sen University (O2022052).

### EAU Model Establishment and Severity Evaluation

Mice were immunized with a mixture containing 2 mg mL^−1^ of human IRBP_1‐20_ (Shanghai GiL Biochem Ltd.) and complete Freund's adjuvant (Difco, San Jose, CA, USA) which included 2.5 mg of Mycobacterium tuberculosis strain H37Ra. This mixture was prepared at a 1:1 volume ratio. Additionally, on days 0 and 2 post‐immunization, mice received intraperitoneal injections of 0.25 µg pertussis toxin diluted in phosphate‐buffered saline (PBS). The severity of experimental EAU in the mice was assessed blindly using ophthalmoscopic examination and histopathology as previously reported. The clinical scoring of EAU via fundoscopy ranged from 0 to 4, based on observed retinal infiltration and vasculitis. After euthanasia, mouse eyeballs were harvested, fixed, dehydrated, embedded in paraffin, and sectioned into 4 µm slices. These sections were stained with hematoxylin and eosin (H&E) and subsequently scanned for pathological scoring.

### Treatment Protocols

GSK484 was dissolved in 4% dimethylsulfoxide, 30% polyethylene glycol 300, and 66% PBS solution. Following this, EAU mice received daily intraperitoneal injections of GSK484 (20 mg kg^−1^, MCE, HY‐100514) or carrier solution for two weeks after EAU immunization. Similarly, EAU mice were also intraperitoneally injected with monoclonal anti‐mouse CD44 (5 mg kg^−1^, Bioxcell, BE0445), anti‐mouse IL‐17A (5 mg kg^−1^, Bioxcell, BE0173) or control IgG antibody every other day for two weeks. On day 14 after immunization, fundus pathological changes were observed, blood was collected using retro‐orbital bleeding, and anatomical dissections were performed to collect retina, CDLN, and spleen samples for subsequent study and analysis.

### DsDNA and MPO Test

Levels of NETs in serum or cell supernatants were assessed by measuring the concentrations of dsDNA and MPO. MPO levels were quantified using an ELISA kit (Abcam, human: ab272101, mouse: ab155458‐1), following the manufacturer's protocol. Briefly, 50 µL of either standards or samples were added to the designated wells, followed by the addition of 50 µL of antibody mix and 100 µL of substrate solution to all wells. Optical density was measured at 450 nm using a full‐wavelength reader (MD SpectraMax Plus 384). For dsDNA quantification, the Quant‐iT PicoGreen dsDNA assay kit (Invitrogen, P7589) was utilized according to the manufacturer's instructions. Pure dsDNA standards and samples were co‐incubated with 100 µL of Quant‐iT PicoGreen reagent for 5 min. Fluorescence was subsequently measured on a TECAN multifunctional reader, using filter settings of 485 nm (excitation) and 538 nm (emission).

### Real‐time Quantitative Polymerase Chain Reaction

In this study, total RNA from neutrophils was extracted using TRIzol Reagent (15596026, Invitrogen) according to the manufacturer's protocol and quantified with a Nanodrop 2000 (Thermo). Real‐time PCR was performed using the ABI Prism 7500 Fast Sequence Detection System (Applied Biosystems) and ChamQ SYBR qPCR Master Mix (Q311‐02, Vazyme). The gene‐specific primer sequences used were listed in Table S2, Supporting Information.

### Flow Cytometry

After isolating lymph nodes and spleens from mice, cells were obtained via homogenization and stimulated at 37 °C for 5 h with 5 ng mL^−1^ phorbol 12‐myristate 13‐acetate (PMA) (Sigma‐Aldrich, St. Louis, MO, USA), 1 µg mL^−1^ brefeldin A (Sigma‐Aldrich), and 500 ng mL^−1^ ionomycin (Sigma–Aldrich). Post‐stimulation, mouse cells were stained with live/dead cell dyes, and surface antibodies, followed by permeabilization, fixation, and intracellular antibody staining. Similarly, human blood samples were collected using Lymphocyte Separation Medium (Stemcell) and stained using a comparable approach.

For mouse samples, cells were stained with APC‐anti‐CD44 (Biolegend, 103012), Brilliant Violet 650‐anti‐IL‐17A (Biolegend, 506930), PerCP/Cyanine5.5‐anti‐CD4 (Biolegend, 100434), PE‐anti‐IFN‐γ (Biolegend, 505808), FITC‐anti‐FOXP3 (ThermoFisher, 11‐5773‐82), PE/Cyanine7‐anti‐GMCSF (Biolegend, 505411), PE‐anti‐CXCR4 (Biolegend, 146506), APC anti‐Ly6G (Biolegend, 127613) and FITC‐anti‐CD11b (Biolegend, 101206), Alexa Fluor 647‐anti‐IRF4 (BioLegend, 646407), Brilliant Violet 421‐anti‐STAT3 (BioLegend, 651009), PE‐anti‐RORγT (BD Biosciences, 562607). For human samples, cells were stained with PE/Cyanine7‐anti‐CD3 (Biolegend, 300419), Brilliant Violet 605‐anti‐CD8a (Biolegend, 301039), FITC‐anti‐IL‐17A (Biolegend, 512304), and PerCP/Cyanine5.5‐anti‐GM‐CSF (Biolegend, 502312). Stained cells were collected using flow cytometry, and the results were analyzed using FlowJo software version 10.0.7 (BD Biosciences).

### Visualisation and Quantification of NETs

Isolated neutrophils (1 × 10^6^ cells mL^−1^) were seeded on cell climbing slices coated with poly‐D‐lysine in 12 well plates and allowed to adhere for 30 min before stimulation with PMA (50 nm) for 5 h at 37 °C and 5% CO2, and then fixed with paraformaldehyde for 30 min. After 1 h blocking with PBS with 3% goat serum and 1% Bovine Serum Albumin (BSA), NETs were detected using anti‐MPO (R&D, AF3667‐SP), anti‐histone H3 (Abcam, ab281584), diluted 1/100 in blocking buffer for 2 h at 37 °C. Slices were then incubated with 1/1000 Alexa Fluor 555 donkey anti‐goat antibody (Abcam, ab150130) and Alexa Fluor 488 goat anti‐rabbit antibody (proteintech, SA00013‐2) for 2 h at 37 °C. DNA was stained with 4′,6‐diamidino‐2‐phenylindole (DAPI) (Fluoroshield Mounting Medium with DAPI, Abcam). NETs were visualized by using Digital Pathology Slide Scanner (KF‐PRO‐020, Ningbo Jiangfeng Medical Technology) and blindly quantified using Image J software. Images were evaluated for MPO and DNA containing; nuclear phenotypes and NETs were counted and expressed as a percentage of the total number of cells in the fields.

### Immunohistochemistry, Immunofluorescence, and Multiplex Immunofluorescence

To assess the expression of specific proteins, IHC and MIF were performed on paraffin‐embedded mouse lymph nodes and spleen specimens. Following deparaffinization with an eco‐friendly clearing agent, the slides were rehydrated in alcohol. Endogenous peroxidase activity was quenched with 10% hydrogen peroxide, followed by microwave‐assisted antigen retrieval. For IHC, slides were incubated with anti‐CD44 (proteintech, 65117‐1‐Ig) at 4 °C for 16 h. After washing off the primary antibody, an enzyme‐labeled goat anti‐mouse/rabbit IgG polymer was applied and incubated for 1 h, followed by DAB staining and hematoxylin counterstaining. For IF, slides were incubated with anti‐MPO (R&D, AF3667‐SP), and anti‐histone H3 (Abcam, ab281584) for 2 h at 37 °C to identify the expression level of NETs in lymph nodes and spleen specimens. Slides were then incubated with 1/1000 Alexa Fluor 555 donkey anti‐goat antibody (Abcam, ab150130) and Alexa Fluor 488 goat anti‐rabbit antibody (proteintech, SA00013‐2) for 2 h at 37 °C.

MIF was conducted according to the manufacturer's instructions for the multiplex immunofluorescence kit (Panovue,10002100100). Specifically, after antigen retrieval, primary antibodies were incubated at room temperature for 1 h, followed by the addition of an HRP‐conjugated secondary antibody solution. Fluorescent staining was then achieved using a signal amplification fluid diluted with a fluorescent dye. After staining one antibody, a new round of antigen retrieval, primary and secondary antibody incubation, and fluorescent dye application were performed. Primary antibodies used included anti‐myeloperoxidase (R&D, AF3667‐SP), anti‐histone H3 (Abcam, ab281584), anti‐CD44 (proteintech, 65117‐1‐Ig), anti‐CD4 (proteintech, 67786‐1‐Ig), and anti‐IL‐17A (proteintech, 66148‐1‐Ig). Nuclei were counterstained with DAPI, and slides were mounted using an anti‐fading mounting medium. Stained slides were scanned, and IHC scores or counts of positive cells were quantified using HALO software (Indicalab, New Mexico, USA). In the HALO software analysis, IHC results were quantified based on the automatically generated IHC scores, which were primarily determined by the staining intensity and the proportion of positive cells (Figure S16A, Supporting Information). IF results were quantified by the proportion of positive cells relative to the total cell count (Figure S16B, Supporting Information).

### Isolation of CDLNs

CDLNs were harvested from mice and mixed with RPMI‐1640 medium containing 2% fetal bovine serum (FBS, Thermo Fisher Scientific), penicillin/streptomycin (Thermo Fisher Scientific), 3 mg mL^−1^ collagenase IV (Sigma‐Aldrich), and 40 mg mL^−1^ DNase I (Sigma‐Aldrich). The mixture was then incubated at 37 °C for 15 min. Following incubation, cells were pooled, and the digested cells were collected and filtered through a 70 µm cell strainer. The resulting single‐cell suspension contained 1 × 10^7^ cells mL^−1^ with viability of ≥85%, as determined by the Countess II automated cell counter (Thermo Fisher Scientific).

### Isolation of PBMC

Blood was drawn from healthy donors using anticoagulant tubes and then diluted twofold before adding a lymphocyte separation medium (Stemcell). PBMCs were isolated using density gradient centrifugation. For in vitro culture, PBMC were maintained in X‐VIVO medium (LONZA) supplemented with 10% FBS, interleukin‐2 (100 IU mL^−1^, Miltenyi Biotec.), and ImmunoCult™ Human CD3/CD28 T Cell Activator (10 µL mL^−1^, InvivoGen). PBMC from mice were obtained by collecting blood through ocular puncture using EDTA‐coated tubes, followed by multiple rounds of red blood cell lysis.

### Isolation of Neutrophils

Following euthanasia, mouse femurs were isolated, and after removal of the femoral heads, bone marrow cells were flushed out using a syringe and filtered through a 70 µm cell strainer to obtain a suspension of myeloid cells. The resultant cell suspension was subjected to red blood cell lysis, followed by neutrophil isolation using a mouse neutrophil isolation kit (Stemcell, 19762) as per the manufacturer's instructions. Specifically, the myeloid cell suspension was sequentially treated with an enrichment cocktail, biotin selection cocktail, and magnetic particles, and finally, mouse neutrophils were enriched using a magnetic separator. Blood was drawn from healthy human donors, and human neutrophils were isolated using a human neutrophil isolation kit (Stemcell, 19666), following a protocol similar to that used for the mouse cells.

### Induction and Extraction of NETs

Neutrophils at a concentration of 5 × 10^6^ cells mL^−1^ were stimulated with 50 nm PMA and incubated at 37 °C with 5% CO2 for 4 h to induce Netosis. After stimulation, the supernatant was gently aspirated, and each well was thoroughly washed with 4 °C cold PBS. The wash fluid was then collected and centrifuged at 450 g for 10 min at 4 °C to remove any residual cells. The supernatant was subsequently centrifuged at 18000 g for 10 min at 4 °C to precipitate all NETs. The concentration of dsDNA was measured to determine the level of enriched NETs. In the in vitro experiment, NETs were pre‐treated with DNase I (5 µg mL^−1^, MCE, HY‐108882) and Proteinase K (25 µg mL^−1^, MCE, HY‐108717) to identify which specific component of NETs mediates the expression of CD44 and IL‐17A.

### In Vitro Co‐Culture and Adoptive Transfer Experiments

After isolating CDLN cells from EAU mice, these cells were cultured in vitro using RPMI‐1640 complete medium supplemented with 10% FBS and 1% penicillin/streptomycin. Cells were stimulated with IRBP_1‐20_ (20 µg mL^−1^) and subsequently, in the experimental group, either isolated NETs were added, while an equivalent volume of PBS was added to the control group. Cultures were maintained for 72 h before performing FCM.

For the adoptive transfer experiment, CD4^+^ T cells isolated from CDLN and spleen cells were co‐cultured with NETs for 72 h, then washed three times with PBS and intravenously injected into wild‐type C57BL/6J mice (2 × 10^7^ cells per mouse). Two weeks later, the development of EAU was assessed using fundoscopy. The specific procedure of the mouse adoptive transfer experiment was described in Figure S3 (Supporting Information), consistent with previous studies.^[^ ^21^, ^24^, ^64^, ^65^ ^]^

### ScRNA‐seq Data Processing

The scRNA‐seq libraries were prepared using the Chromium Single Cell 5′ Library and Gel Bead Kit (10x Genomics, 120237) according to the manufacturer's instructions. Specifically, cells were washed in a 0.04% BSA buffer (0.02 g BSA dissolved in 50 mL of deionized PBS) and subsequently encapsulated in droplets. This was followed by stepwise reverse transcription, emulsion breakup, barcode‐cDNA purification with Dynabeads, and PCR amplification. The amplified cDNA was then used for constructing the 5′ gene expression libraries. This process included fragmentation and end repair, double size selection using SPRIselect beads, and sequencing of 50 ng amplified cDNA on the NovaSeq platform (Illumina NovaSeq6000), generating 150 bp paired‐end reads. Initial processing of the sequencing data was performed using the CellRanger software v3.0.2 (10x Genomics).

ScRNA‐seq data were integrated and clustered using the “Seurat” package (v3.1) in R (v4.0.0), with batch effects among different samples mitigated by the “Harmony” package (v1.0). Quality control thresholds were set to include cells with gene counts ranging from 300 to 4000 and mitochondrial gene content below 15%. Cells highly expressing Hbb‐a1 and Hbb‐bs were identified as erythrocytes and excluded from further analysis.

### Dimensionality Reduction and Clustering Analysis

Following data quality control, normalization of the dataset was performed using the “NormalizeData” function within the Seurat package in R. Subsequently, cells were clustered using the “FindClusters” function, and data visualization was achieved through the two‐dimensional t‐SNE algorithm via the “RunTSNE” function. Additionally, the “FindAllMarkers” function was employed to identify marker genes for different clusters. Cell types for each cluster were manually determined based on the high‐expression genes of different clusters. DEGs between cell types were generated using the “FindMarkers” function through the Wilcoxon rank‐sum test and adjusted via Bonferroni correction (|Log2(fold change)| > 0.25 and adjusted *p*‐value < 0.05). DEGs used for the volcano plots were available in the supplementary data.

### GO and GSVA Analysis

Gene Ontology analysis was performed using the Metascape online tool (www.metascape.org). The p‐values for GO terms were calculated based on the cumulative hypergeometric distribution via the Metascape tool. Only 5–10 disease‐related GO pathways were displayed for different cell types. GSVA was an unsupervised gene set enrichment method that assesses changes in pathway activity within individual samples. This analysis was conducted using the “GSVA” package (v1.48.3) in R. Results of the pathway analysis were visualized using the pheatmap (v1.0.12) and “ggplot2” packages (v3.2.1).

### Pseudotime Trajectory Analysis

Pseudotime analysis was conducted using the Monocle 2 software package (v2.30.1), designed to arrange single cells based on their progression through biological processes. Cells were ordered along pseudotime trajectories using marker genes identified for distinct clusters through the “FindAllMarkers” function. Ridge plots were utilized to display the patterns of cellular changes across different groups along the pseudotime sequence. In addition, this study employed the scVelo tool to calculate the gene expression velocity vectors for each cell, further elucidating the differentiation dynamics of Th17 cells.^[^ ^66^ ^]^

### Statistics

All experiments were performed at least three times. Data analysis and visualization were conducted using GraphPad Prism software v 8.0.2 (GraphPad, Inc., La Jolla, CA, USA) and R language (Version 4.3.2). Results were presented as mean ± standard error of the mean (SEM). Statistical analyses were carried out using the Mann–Whitney Test, unpaired two‐tailed Student's *t*‐tests, Kruskal‐Wallis test, one‐way analysis of variance (ANOVA), and Bonferroni multiple comparison tests. A p‐value of <0.05 was considered statistically significant, with ns indicating no significant difference, *p < 0.05, **p < 0.01, ***p < 0.001, ****p < 0.0001.

### Data Availability

Human scRNA‐seq data were available in the Genome Sequence Archive (GSA) in the National Genomics Data Center, Beijing Institute for Genome Research (https://ngdc.cncb.ac.cn/gsa‐human/) (GSA Accession No. HRA007776). Mice scRNA‐seq data were deposited under the GSA Accession No. CRA017271.

## Conflict of Interest

The authors declare no conflict of interest.

## Author Contributions

Y.W., K.N., and Z.H. contributed equally to this work. Y.W., K.N., Z.H., and W.S. conceptualized the study and developed the methodology. Y.W., K.N., and Z.H. conducted the experiments and performed the analysis. Y.W., K.N., Z.H. B.C., J.C., Y.W., J.B., H.H., Q.C., and Z.Z. made the visualization. R.J. and W.S. acquired funding and supervised the study. Y.W., K.N., Z.H., and W.S. wrote the original manuscript. All authors read and approved the final version of the manuscript.

## Supporting information



Supporting Information

## Data Availability

The data that support the findings of this study are available from the corresponding author upon reasonable request.
