# Supplementary material for: NETs‐CD44‐IL‐17A Feedback Loop Drives Th17‐Mediated Inflammation in Behçet's Uveitis
Source: Adv Sci (Weinh). 2025 Feb 27;12(16):2411524. doi: 10.1002/advs.202411524 (PMC12021058; doi:10.1002/advs.202411524)
Supplement: Supplementary file 1 — Supporting Information [file ADVS-12-2411524-s001.docx]

**
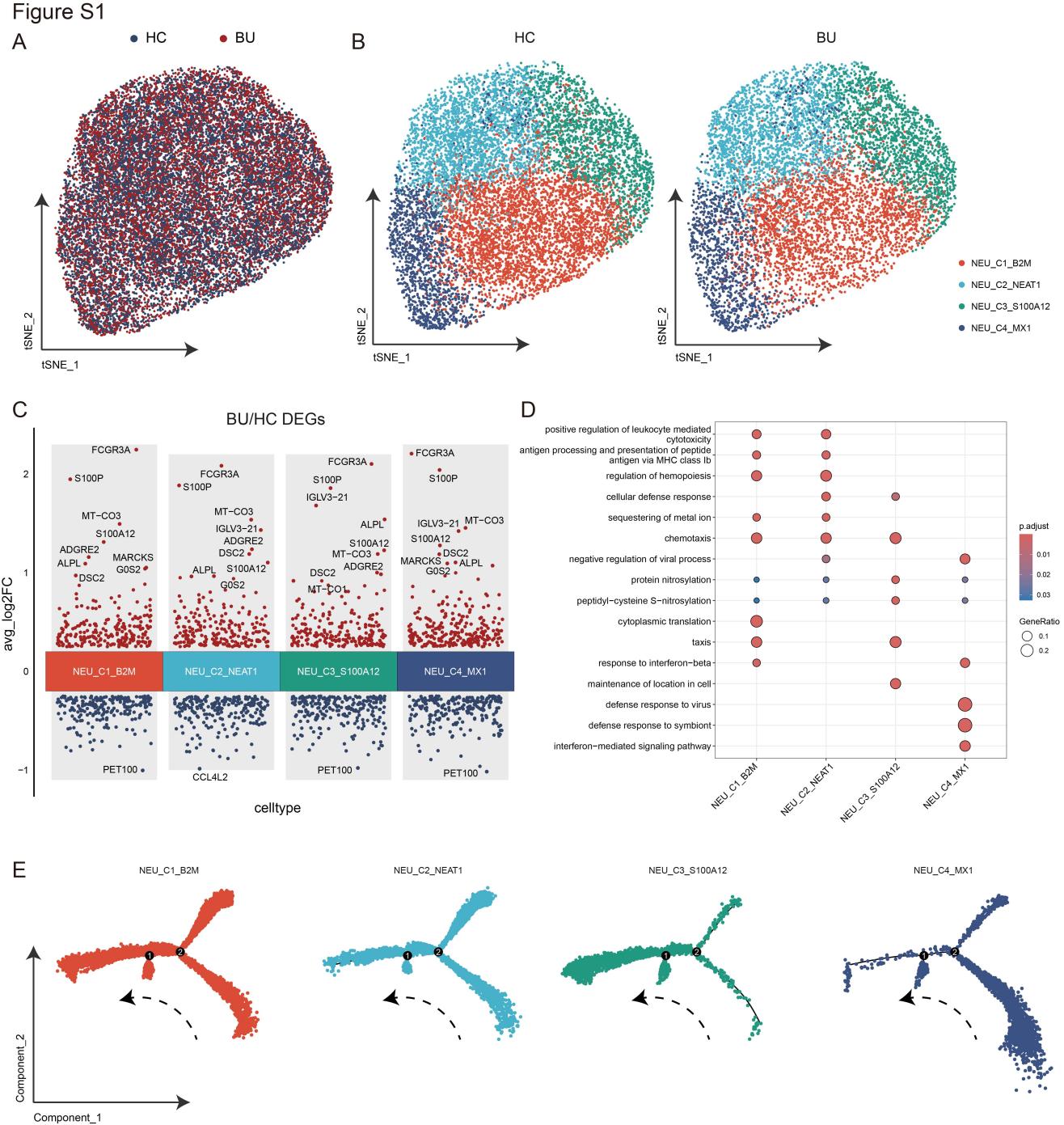
**

**Figure S1. Clusters, differentially expressed genes, GO analysis and pseudotime trajectory analysis of neutrophil subsets in HC and BU patient.**

**A.** TSNE plot of total neutrophil in HC and BU patient.

**B.** TSNE plot showing the clusters of neutrophil subsets in HC and BU patient.

**C.** Volcano plot showing the up- and down-regulated DEGs of neutrophil subsets in BD/HC comparison group. Red and blue plots indicate up- and down-regulated DEGs of neutrophil subsets, respectively.

**D.** Representative GO terms and pathways enriched in top 100 highest-expressed genes in neutrophil subsets.

**E.** Pseudotime trajectory analysis of neutrophil subsets.


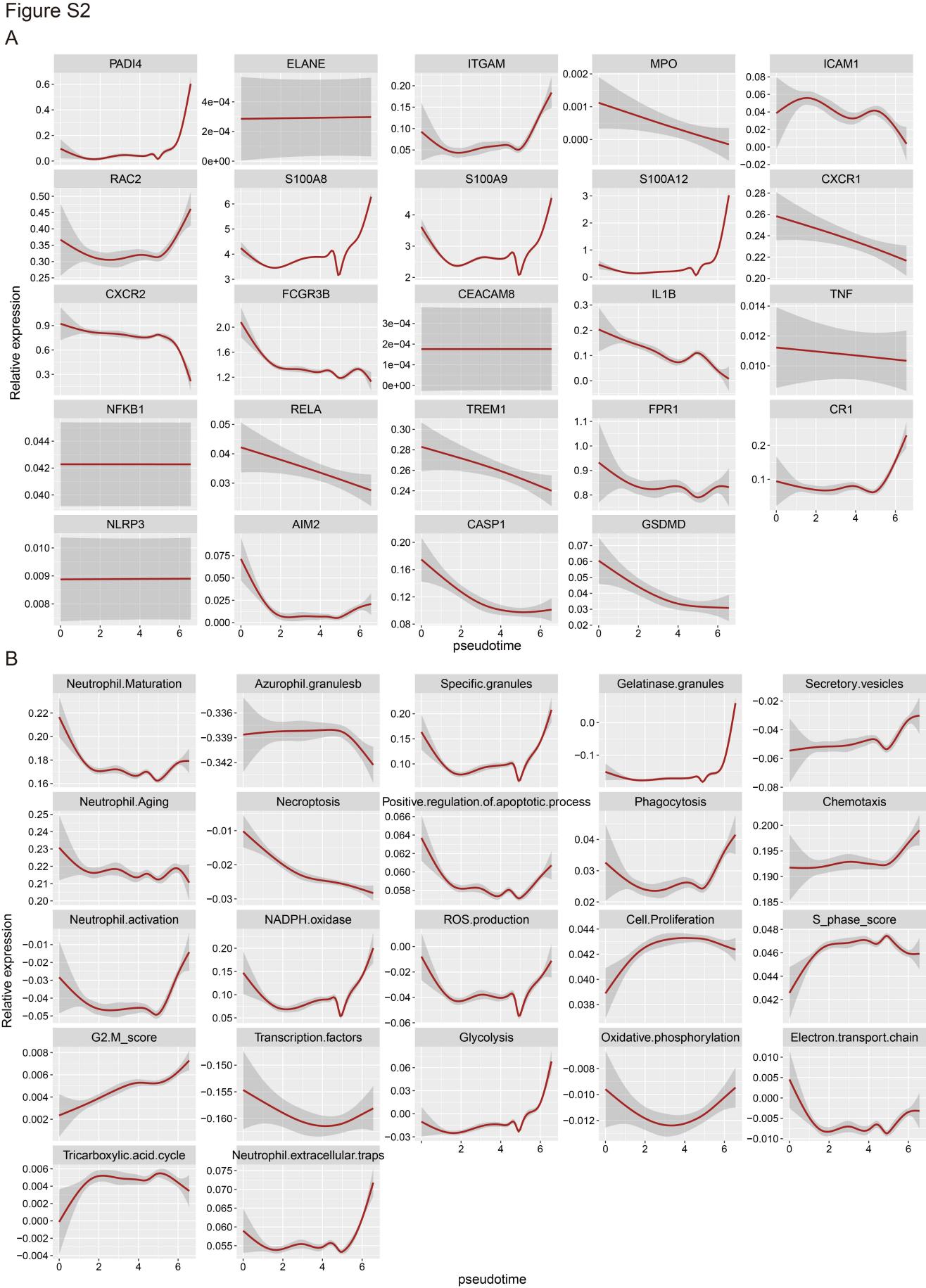


**Figure S2. Expression transition of key genes and function of neutrophil along the pseudotime.**

**A.** Expression transition of key genes associated with neutrophil function and NETs along the pseudotime.

**B.** Expression transition of neutrophil function along the pseudotime.


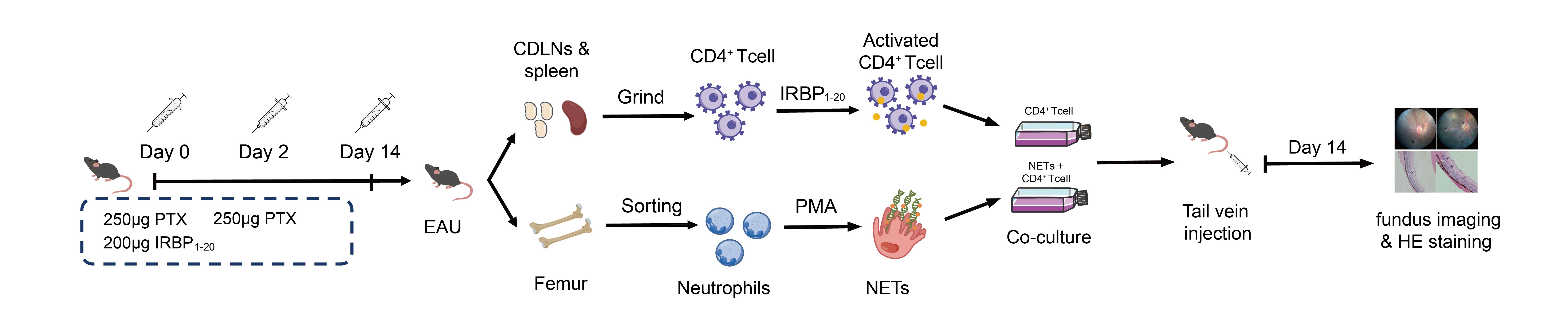


**Fig.S3 Schematic illustration of in vitro and adoptive transfer experiments**

**

**

**Figure S4. FCM analysis of in vitro and in vivo experiments after GSK484 treatment.**

1. The expression of CD44 and CXCR4 of CD4^+^ T cells in PBMC by FCM analysis (n = 5/group).
2. The expression of CD44, CXCR4 and GM-CSF of CD4^+^ T cells in CDLNs and spleen of EAU and GSK484 groups by FCM analysis (n = 5/group).
3. The mean fluorescence intensity of transcription factors (RORγt, STAT3, IRF4) of CD4^+^ T cells in CDLNs and spleen of EAU and GSK484 groups by FCM analysis (n = 5/group).
4. FCM analysis of CD44 and IL-17A expression on CD4^+^ T cells co-cultured with untreated NETs, Proteinase K-treated NETs or DNaseI-treated NETs in vitro (n = 5/group).

Significance in **A, B, D** was determined using one-way ANOVA and Bonferroni multiple comparison test. Significance in **C** was determined using unpaired two-tailed Student's t-tests. Results are presented as mean ± SEM, with ns indicating no significant difference, *p < 0.05, **p < 0.01, ***p < 0.001, ****p < 0.0001.

**

**

**Figure S5. FCM analysis of in vitro and in vivo experiments in PADI4-KO mouse.**

**A-B.** Fundus images, clinical scores, H&E staining, and pathological scores of EAU and PADI4-KO groups (n = 5/group).

**C.** FCM analysis of the proportion of infiltrating CD4^+^ T cells in the retina of two groups (n = 5/group).

**D.** Serum levels of dsDNA and MPO in EAU and PADI4-KO groups (n = 5/group).

**E-F.** FCM analysis of IL-17A, IFN-γ and FOXP3 expression in CD4^+^ T cells derived from CDLNs and spleen of EAU and PADI4-KO groups (n = 5/group).

**G-H.** FCM analysis of CXCR4 and CD44 expression in CD4^+^ T cells derived from CDLNs and spleen of two groups (n = 5/group).

Significance in **A-B** was determined using Mann-Whitney Test. Significance in **C-H** was determined using unpaired two-tailed Student's t-tests. Results are presented as mean ± SEM, with ns indicating no significant difference, *p < 0.05, **p < 0.01, ***p < 0.001, ****p < 0.0001.

**
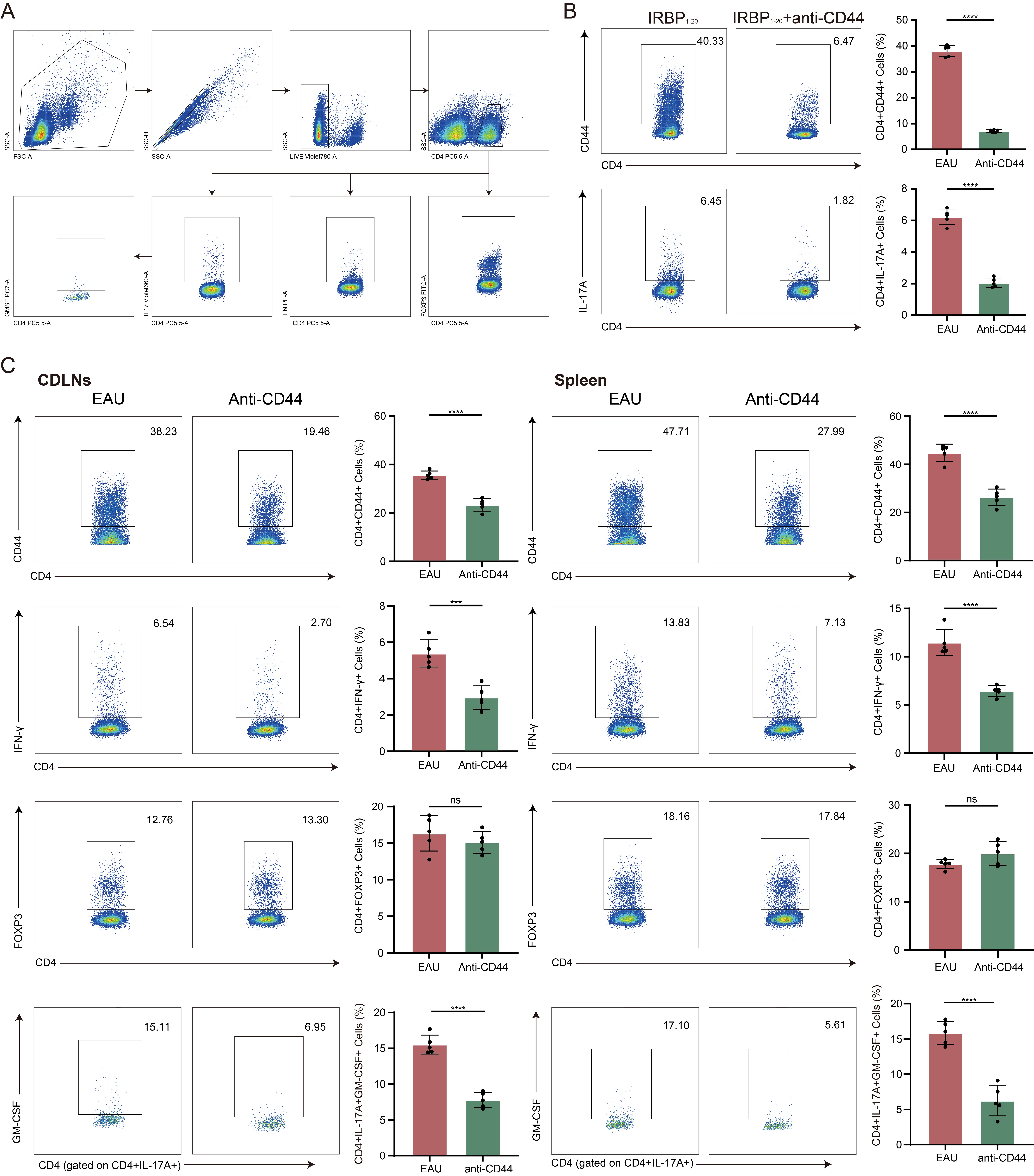
**

**Figure S6. FCM analysis of in vitro and in vivo experiments after anti-CD44 treatment.**

**A.** Gating strategy of Th17, Th1, Treg and GM-CSF^+^ Th17 cells

**B.** The expression levels of CD44 and IL-17A in CD4^+^ T cells by FCM analysis with and without anti-CD44 treatment (1μg/ml) for 72 hours in vitro (n = 5/group).

**C.** The expression levels of CD44, IFN-γ, FOXP3 and GM-CSF in CD4^+^ T cells by FCM analysis in CDLNs and spleen between EAU and anti-CD44 treatment groups (n = 5/group).

Significance in **B-C** was determined using unpaired two-tailed Student's t-tests. Results are presented as mean ± SEM, with ns indicating no significant difference, *p < 0.05, **p < 0.01, ***p < 0.001, ****p < 0.0001.

**
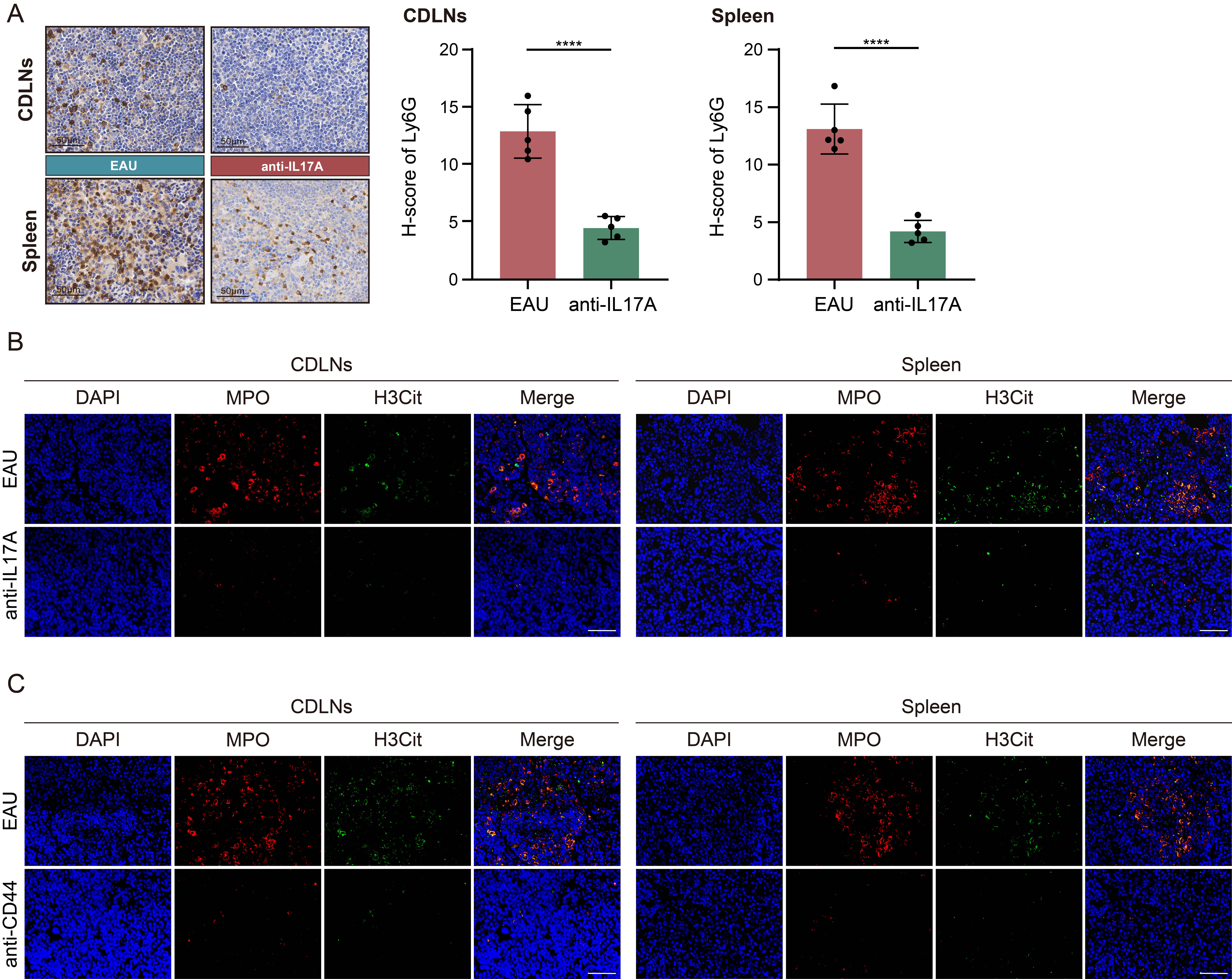
**

**Figure S7. IHC and IF assessment in CDLNs and spleen between EAU and anti-CD44 /anti-IL-17A treatment groups.**

1. IHC showing the expression level of Ly6G in CDLNs and spleen between EAU and anti-IL-17A treatment groups (n = 5/group).
2. IF assessment of NETs levels in CDLNs and spleen between EAU and anti-IL-17A treatment groups.
3. IF assessment of NETs levels in CDLNs and spleen between EAU and anti-CD44 treatment groups.

Significance in **A** was determined using unpaired two-tailed Student's t-tests. Results are presented as mean ± SEM, *p < 0.05, **p < 0.01, ***p < 0.001, ****p < 0.0001.

**
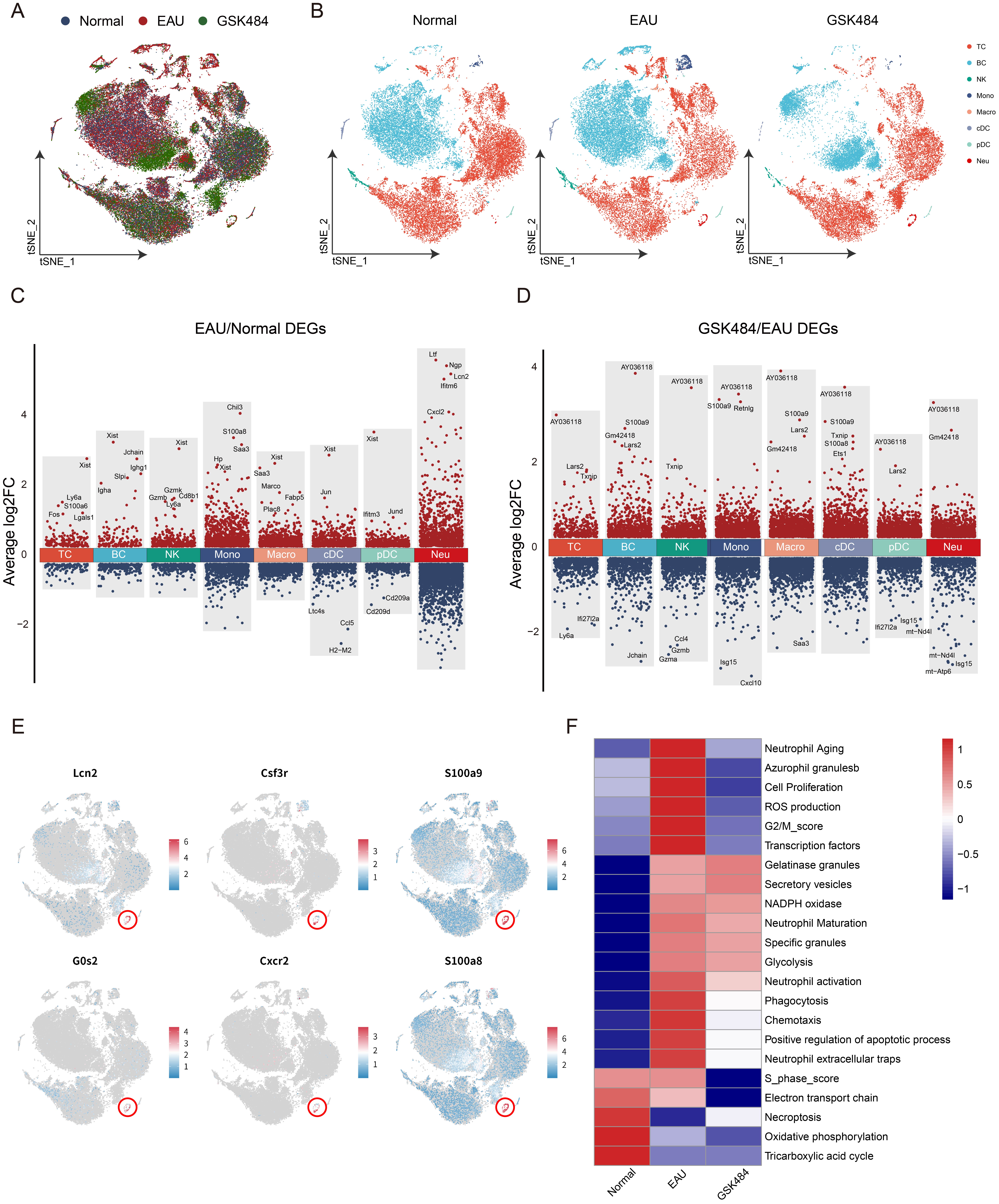
**

**Figure S8. The DEGs of immune cell subsets and GSVA analysis of neutrophil in normal, EAU and GSK484 groups.**

1. TSNE plot of total T cells of CDLNs in three groups.

**B.** TSNE plot showing clusters of immune cell subsets in normal, EAU and GSK484 groups.

**C-D.** Volcano plot showing the up- and down-regulated DEGs of immune cell subsets in EAU/normal (C) and GSK484/EAU (D) comparison group. Red and blue plots indicate up- and down-regulated DEGs of immune cell subsets, respectively.

**E.** TSNE plots of canonical markers for neutrophil subsets.

**F.** GSVA analysis of neutrophil in three groups.


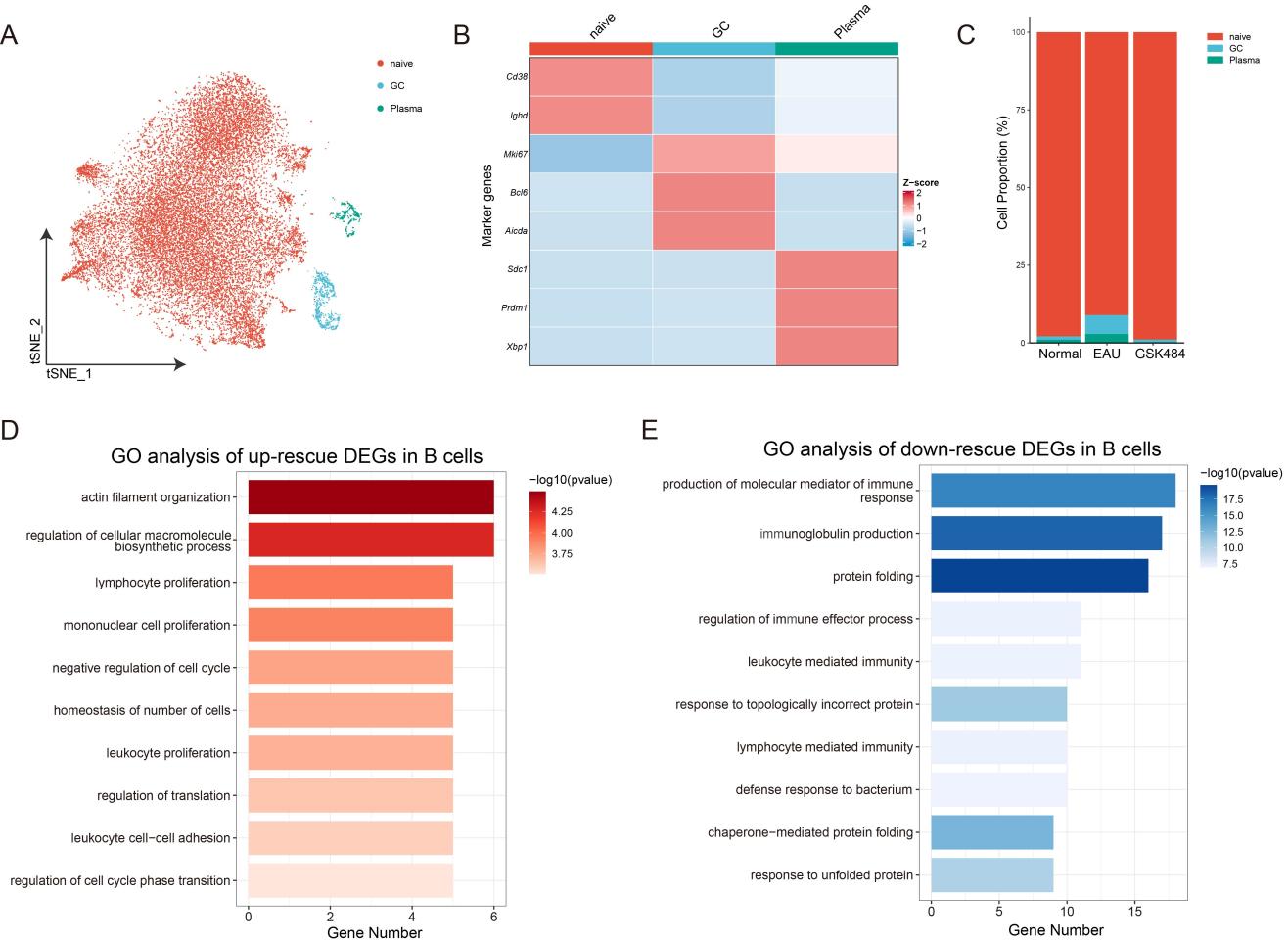


**Figure S9. The change of cell proportion and signaling pathways of B cell subsets in normal, EAU and GSK484 groups.**

**A.**TSNE plot of total B cells of CDLNs in three groups.

**B.** Heatmap showing scaled expression of discriminative gene sets for B cell subsets in CDLNs.

**C.** The proportions of different B cell subsets in three groups.

**D-E.** Representative GO terms and pathways enriched in up- (D) and down-(E) rescue DEGs in B cells.


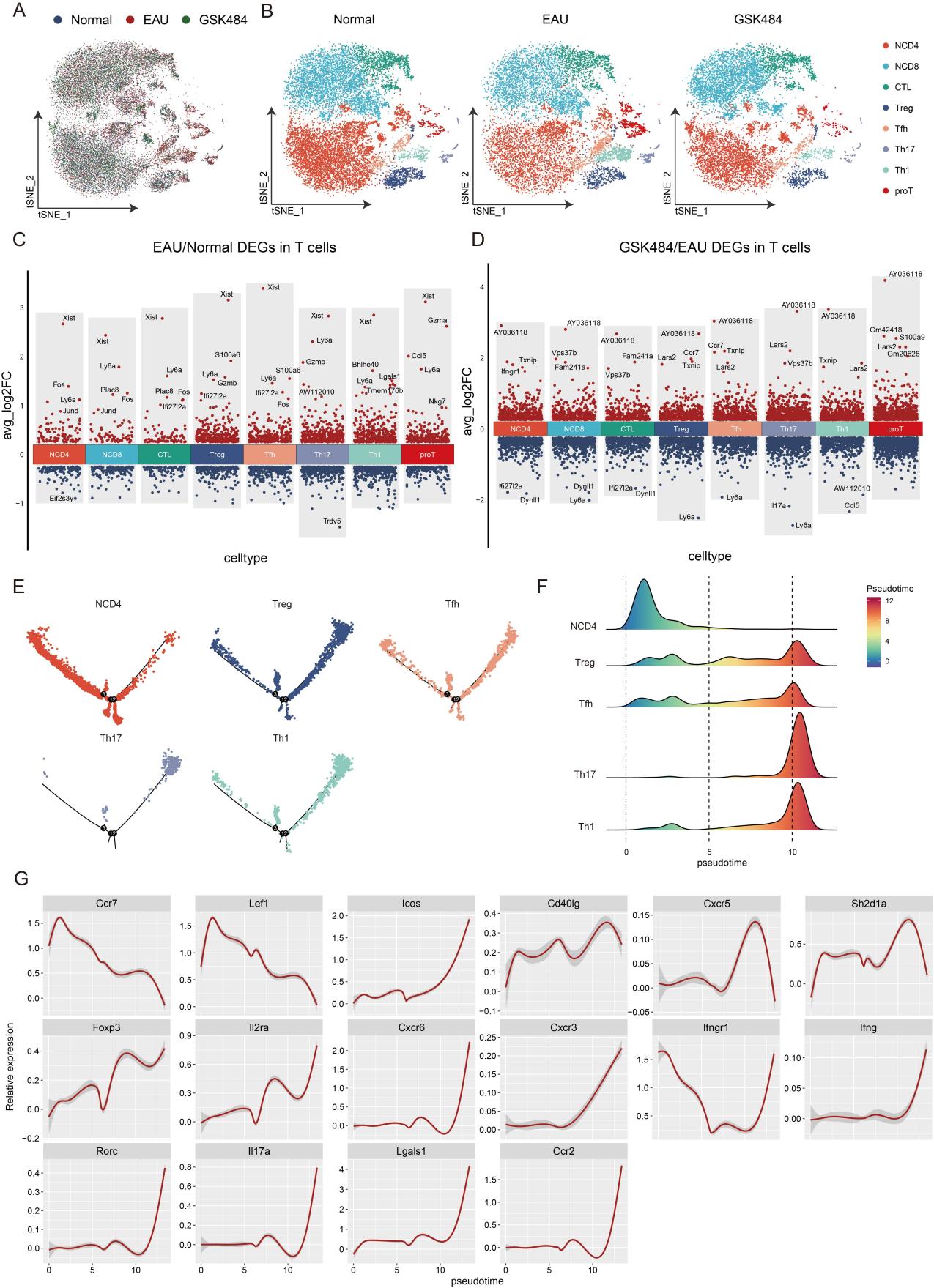


**Figure S10. The DEGs of T cell subsets and pseudotime trajectory analysis of CD4^+^ T cells subsets in normal, EAU and GSK484 groups.**

1. TSNE plot of total T cells of CDLNs in normal, EAU and GSK484 groups.

**B.** TSNE plot showing clusters of T cell subsets in normal, EAU and GSK484 groups

**C-D.** Volcano plot showing the up- and down-regulated DEGs of T cell subsets in EAU/normal (C) and GSK484/EAU (D) comparison group. Red and blue plots indicate up- and down-regulated DEGs of immune cell subsets, respectively.

**E.** Pseudotime trajectory analysis of CD4**^+^** T cells subsets.

**F.** Ridge plot of CD4^+^ T cell proportions along the pseudotime among three groups.

**G.** Expression transition of marker genes of CD4**^+^** T cells subsets along the pseudotime.

**
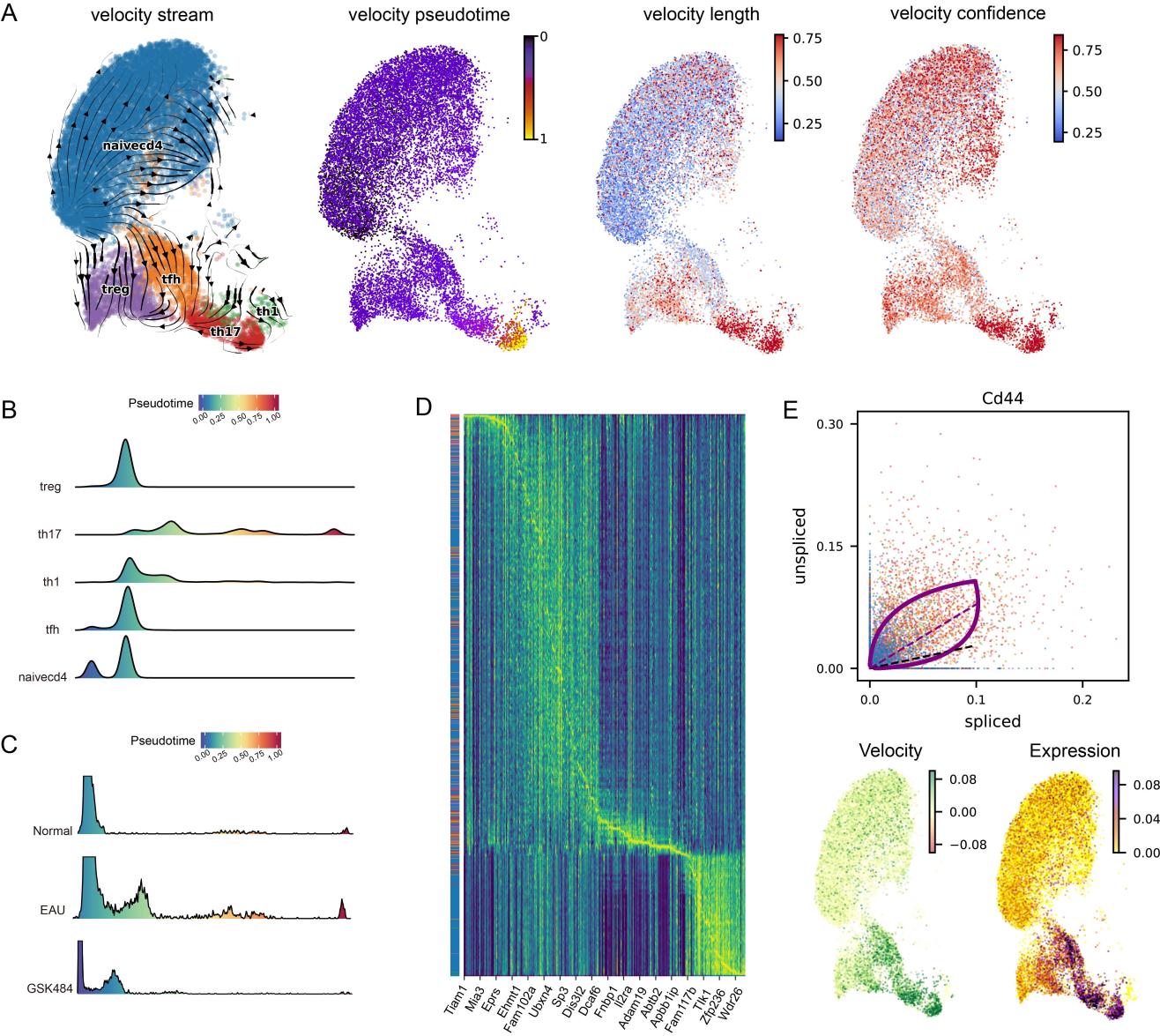
**

**Figure S11. RNA velocity analysis of CD4^+^ T cell subsets in the normal, EAU, and GSK484 groups.**

**A.** Visualization of RNA velocity analysis, including velocity streams, pseudotime, length, and confidence.

**B.** Distribution of RNA velocity pseudotime across different groups, including normal, EAU, and GSK484 groups.

**C.** Distribution of RNA velocity pseudotime across different cell types, including NCD4, Treg, Tfh, Th17, and Th1 cells.

**D.** Genes exhibiting the most significant dynamic behaviors in the RNA velocity analysis.

**E.** RNA velocity analysis of the *Cd44* gene.

**
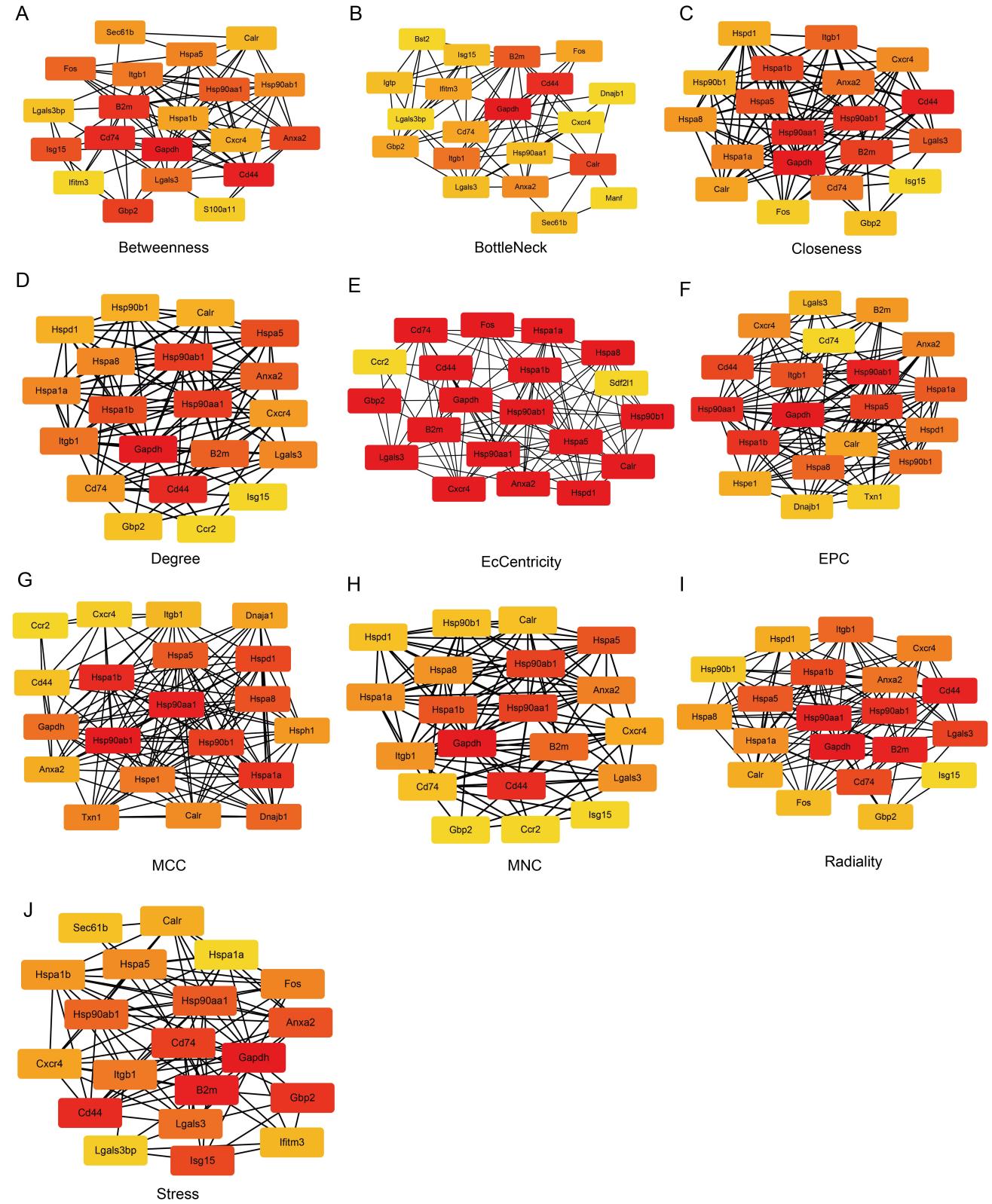
**

**Figure S12. Six core genes from the top 20 hub genes were identified using the cytoHubba plugin with ten algorithms.**

**A-J.** Six core hub genes, namely, Cd44, Gapdh, hsp90aa1, Anxa2, Cxcr4, and Calr from the top 20 hub genes of down-rescue DEGs in CD4^+^ T cells, were identified using the cytoHubba plugin with ten algorithms: Betweenness (A), BottleNeck (B), Closeness (C), Degree (D), EcCentricity (E), EPC (F), MCC (G), MNC (H), Radiality (I) and Stress (J).**
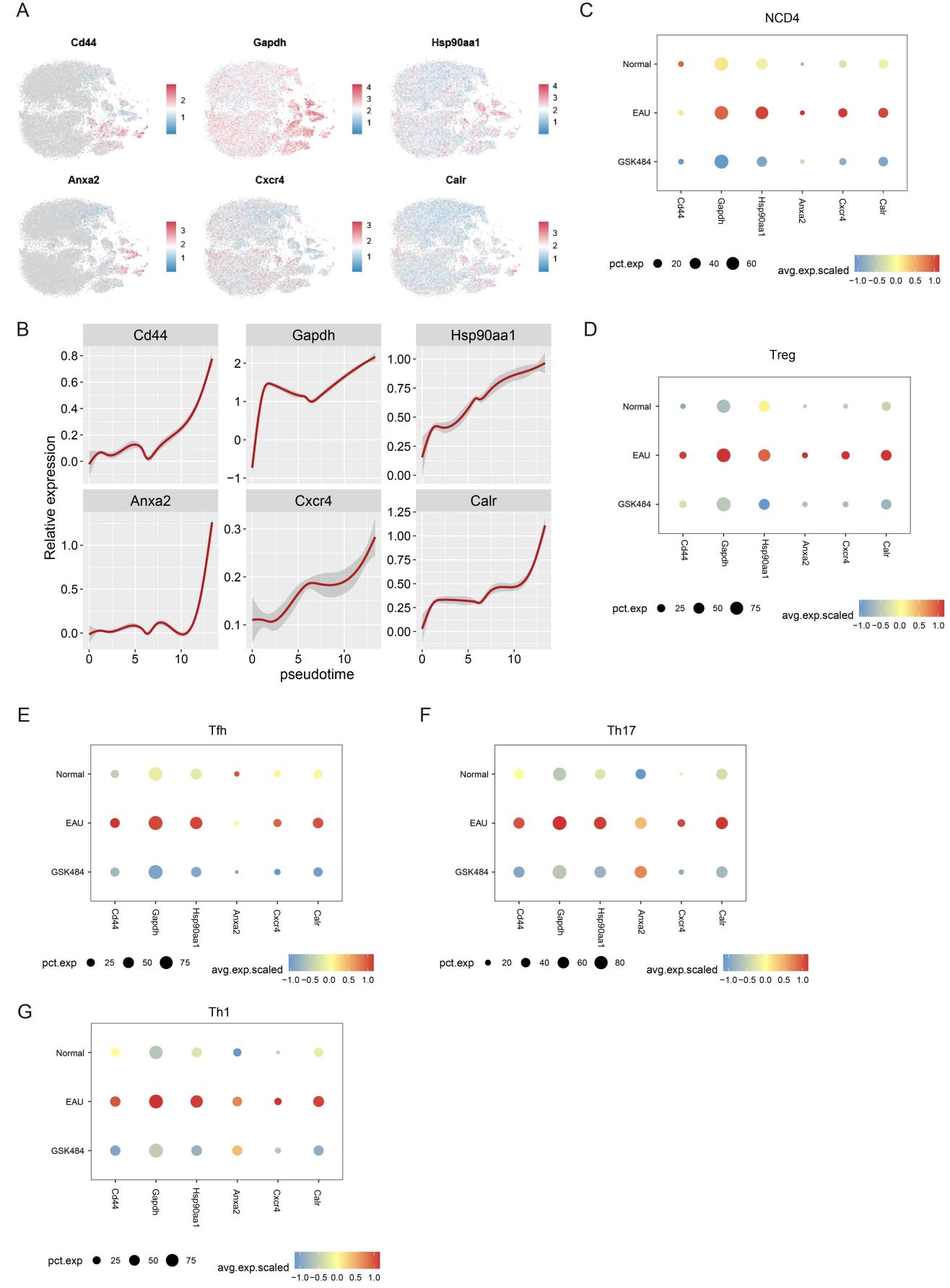
**

**Figure S13. Pseudotime trajectory analysis of six core genes and their expression level in all CD4^+^ T cell subsets in normal, EAU and GSK484 groups**

1. TSNE plots of six core genes identified using the cytoHubba plugin with ten algorithms.
2. Expression transition of six core genes along the pseudotime.

**C-G.** Dot plot showing the expression levels of six hub genes in NCD4 (C), Treg (D), Tfh (E), Th17 (F) and Th1 (G) cells among three groups.





**Figure S14. Analysis of Cell-Cell communication between neutrophils and CD4^+^ T cell subsets in different groups**

1. The number and strength of communications between neutrophils and CD44^high^CD4^+^ T cells, as well as CD44^high^CD4^+^ T cells, across different groups.
2. The number and strength of communications between neutrophils and CD4^+^ T cell subsets in different groups.

**C-D.** Overall information flow of signaling pathways in different groups.

**E-F.** Upregulated and downregulated paired signals between neutrophils and CD44^high^CD4^+^ T cells in different groups.

**G-H.** Upregulated and downregulated paired signals between neutrophils and Th17 cells in different groups.

**
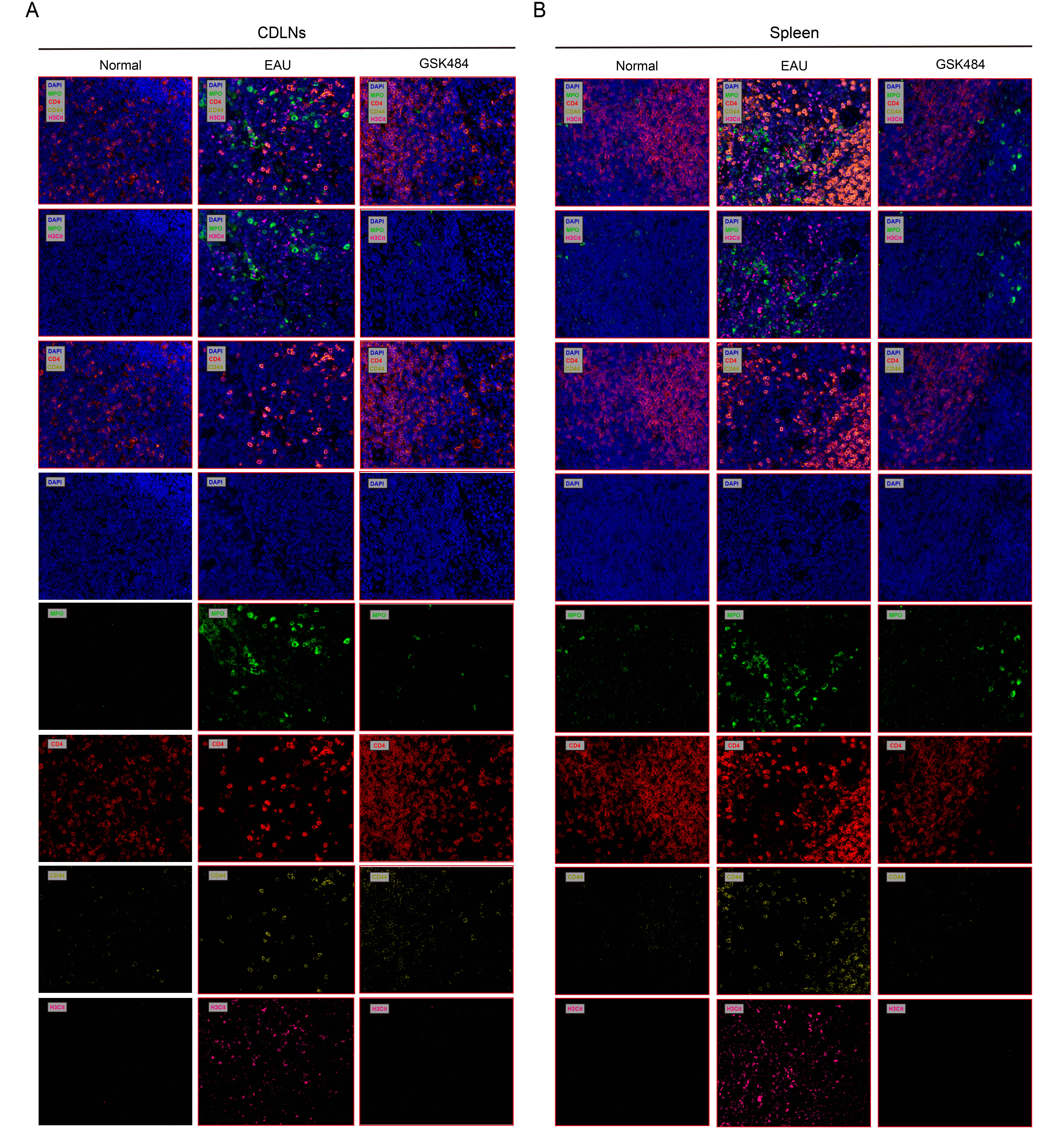
**

**Figure S15. MIF staining demonstrated colocalization of NETs (MPO^+^H3Cit^+^) with CD44^high^CD4^+^ T cells in the EAU group**

**A-B.** MIF staining of NETs (MPO^+^H3Cit^+^) with CD44^high^CD4^+^ T cells in the CDLNs (A) and spleen (B) of EAU group.

**
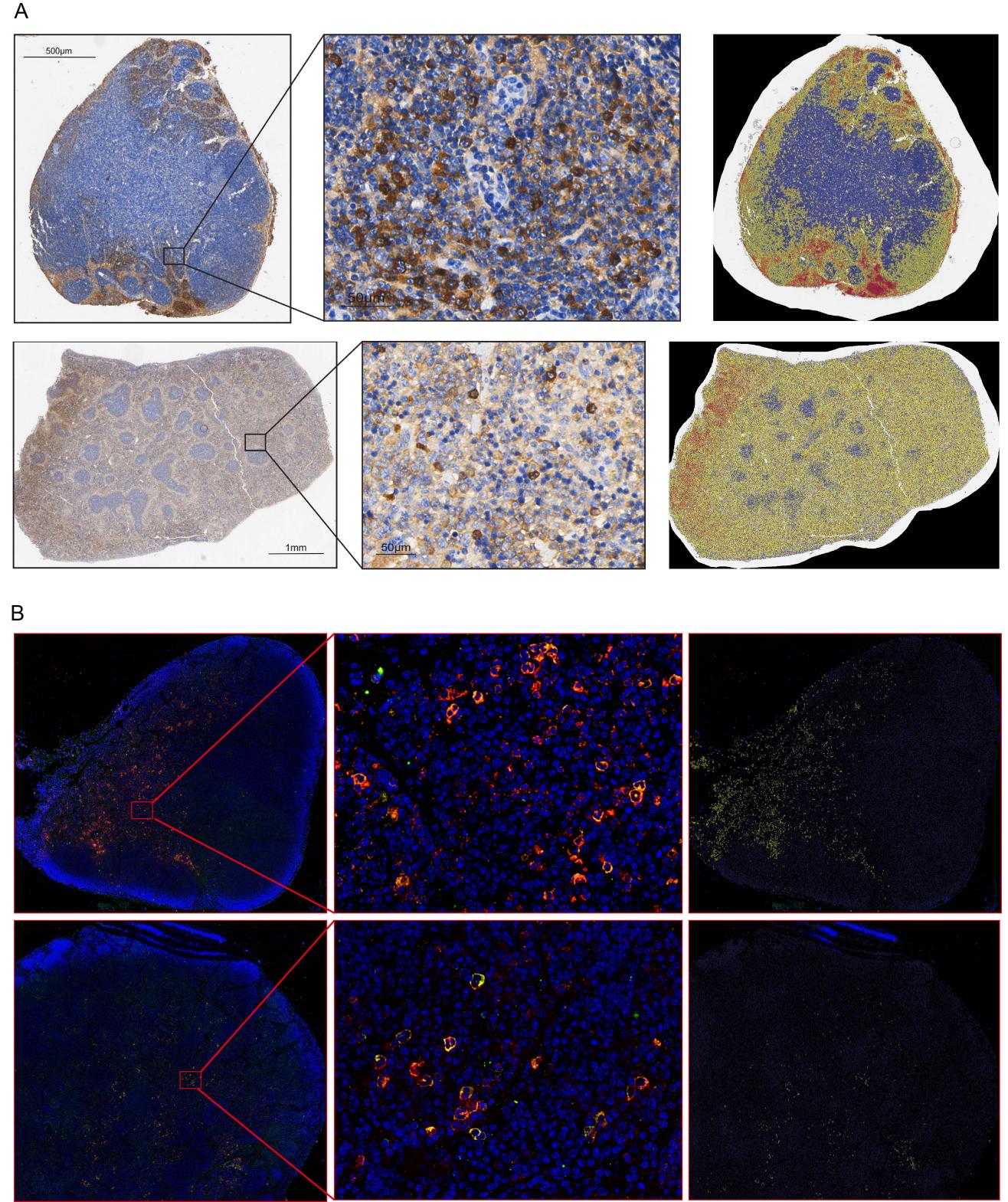
**

**Figure S16. The IHC and IF expression was quantified using by HALO software.**

**A-B.** The example of quantification of IHC (A) and IF (B) stained images by HALO software.

**Table S1.** Individual data of BD patients and HC included in the study.

| No. | Age | Sex | Anterior chamber cells | Anterior chamber flare | Retinal vascular leakages by FFA | Treatment |
| --- | --- | --- | --- | --- | --- | --- |
| BU1 | 22 | Female | 2 | 1 | + | - |
| BU2 | 32 | Male | 1 | 1 | + | - |
| BU3 | 41 | Male | 4 | 2 | + | - |
| BU4 | 25 | Male | 3 | 2 | + | - |
| BU5 | 44 | Female | 2 | 1 | + | - |
| BU6 | 30 | Female | 1 | 1 | + | - |
| BU7 | 51 | Male | 1 | 1 | + | - |
| BU8 | 37 | Female | 3 | 2 | + | - |
| BU9 | 48 | Male | 4 | 3 | + | - |
| BU10 | 36 | Male | 2 | 2 | + | - |
| HC1 | 22 | Female | - | - | - | - |
| HC2 | 33 | Male | - | - | - | - |
| HC3 | 40 | Male | - | - | - | - |
| HC4 | 23 | Male | - | - | - | - |
| HC5 | 43 | Female | - | - | - | - |
| HC6 | 31 | Female | - | - | - | - |
| HC7 | 50 | Male | - | - | - | - |
| HC8 | 37 | Female | - | - | - | - |
| HC9 | 48 | Male | - | - | - | - |
| HC10 | 36 | Male | - | - | - | - |

FFA: fundus fluorescein angiography

**Table S2.** Primer sequences for qPCR.

|  | Forward sequence | Reverse sequence |
| --- | --- | --- |
| Mouse MPO | 5'-CGTGTCAAGTGGCTGTGCCTAT-3' | 5'-AACCAGCGTACAAAGGCACGGT-3' |
| Mouse PADI4 | 5'-ACGCTGCCTGTGGTCTTTGACT-3' | 5'-ACCTCCAGGTTCCCAAAGGCAT-3' |
| Human MPO | 5'-GAGCAGGACAAATACCGCACCA-3' | 5'-AGAGAAGCCGTCCTCATACTCC-3' |
| Human PADI4 | 5'-GCACAACATGGACTTCTACGTGG-3' | 5'-CACGCTGTCTTGGAACACCACA-3' |
